# Role of tumor microenvironment in ovarian cancer metastasis and clinical advancements

**DOI:** 10.1186/s12967-025-06508-0

**Published:** 2025-05-14

**Authors:** Yang Wang, Na Zhu, Jing Liu, Fang Chen, Yang Song, Yue Ma, Zhuo Yang, Danbo Wang

**Affiliations:** 1 Department of Gynecology, Cancer Hospital of China Medical University, Liaoning Cancer Hospital & Institute, Cancer Hospital of Dalian University of Technology, No.44 Xiaoheyan Road, Dadong District, Shenyang, Liaoning Province 110042 People’s Republic of China; 2 Department of Gynecology, People’s Hospital of Liaoning Province, Shenyang, Liaoning Province 110016 People’s Republic of China; 3 Department of Gynecology and Obstetrics, Shengjing Hospital of China Medical University, No.36, Sanhao Street, Heping District, Shenyang, Liaoning 110004 People’s Republic of China

**Keywords:** Ovarian cancer, Tumor microenvironment, Growth, Metastasis, Immune cells, Stromal cells

## Abstract

Ovarian cancer (OC) is the most lethal gynecological malignancy worldwide, characterized by heterogeneity at the molecular, cellular and anatomical levels. Most patients are diagnosed at an advanced stage, characterized by widespread peritoneal metastasis. Despite optimal cytoreductive surgery and platinum-based chemotherapy, peritoneal spread and recurrence of OC are common, resulting in poor prognoses. The overall survival of patients with OC has not substantially improved over the past few decades, highlighting the urgent necessity of new treatment options. Unlike the classical lymphatic and hematogenous metastasis observed in other malignancies, OC primarily metastasizes through widespread peritoneal seeding. Tumor cells (the “seeds”) exhibit specific affinities for certain organ microenvironments (the “soil”), and metastatic foci can only form when there is compatibility between the “seeds” and “soil.” Recent studies have highlighted the tumor microenvironment (TME) as a critical factor influencing the interactions between the “seeds” and “soil,” with ascites and the local peritoneal microenvironment playing pivotal roles in the initiation and progression of OC. Prior to metastasis, the interplay among tumor cells, immunosuppressive cells, and stromal cells leads to the formation of an immunosuppressive pre-metastatic niche in specific sites. This includes characteristic alterations in tumor cells, recruitment and functional anomalies of immune cells, and dysregulation of stromal cell distribution and function. TME-mediated crosstalk between cancer and stromal cells drives tumor progression, therapy resistance, and metastasis. In this review, we summarize the current knowledge on the onset and metastatic progression of OC. We provide a comprehensive discussion of the characteristics and functions of TME related to OC metastasis, as well as its association with peritoneal spread. We also outline ongoing relevant clinical trials, aiming to offer new insights for identifying potential effective biomarkers and therapeutic targets in future clinical practice.

## Introduction

Currently, Ovarian cancer (OC) is the most deadly and third most common gynecological malignancy worldwide, accounting for up to 5% of female cancer fatalities [1]. As a heterogeneous disease, OC encompasses different molecular biology, histological subtypes, and microenvironmental features, all of which affect treatment response and clinical outcomes [2]. Most deaths are of patients presenting with advanced stage, high grade serous OC (HGSOC) 2, 3 (~ 70%), characterized by aggressiveness, late onset and lack of specificity. About 15% of patients with HGSOC succumb to the disease within the first year, and only 25% survive more than five years after diagnosis [3]. Surgical resection is an effective strategy for cytoreduction of the primary disease and any local metastases that may have begun to appear differentiated from the primary lesion. Despite recent advances in curative operations, platinum-based chemotherapy, immunotherapy and targeted agents have provided compelling breakthroughs in the treatment of refractory tumor types, approximately 80% of patients with HGSOC are diagnosed with clinically unsatisfactory clinical outcomes [2, 4].

HGSOC is suggested to progress from early to late stages over an average of only 2 years [5]. Despite extensive research over the past 30 years, treatment options for OC were not significantly improved, with paclitaxel and carboplatin remaining the primary therapeutic agents [6]. While most patients with HGSOC are responsive to platinum-based chemotherapeutic agents, ~ 75% will experience chemoresistant recurrence [7]. Drug resistance remains the main obstacle for increasing the survival of HGSOC. Although the abdominal cavity sets the stage for OC progression and peritoneal metastasis, many challenges related to recurrence and morbidity remain, since the underlying mechanisms are unclear. These variable results highlight the necessity for further in-depth exploration of tumor and host profiling.

The progression of OC is orchestrated not only by tumor cell heterogeneity but also by dynamic interactions between cancer cells and the surrounding tumor microenvironment (TME). Proliferation, angiogenesis, evasion of immune surveillance, apoptosis inhibition and immune system suppression are intrinsically linked to the TME [8]. The intraperitoneal TME creates favorable conditions for the progression of OC and serves as a major determinant of peritoneal metastasis [9]. Earlier reports indicate that at the time of diagnosis, nearly 70% of patients with OC already have peritoneal metastases [10]. In contrast to other tumors, OC metastases commonly occur in the omentum or peritoneum. The omentum serves as an optimal substrate for OC metastasis due to the intricate nature of the intraperitoneal environment, which facilitates and sustains the metastatic process. While the role of the TME in intraperitoneal metastasis in OC is not well understood, evidence indicates that the interaction between tumor cells and stromal cells facilitates the dissemination of OC within the peritoneal cavity [11], which is the main underlying reason for poor prognosis [12]. The critical role of TME for OC genesis, development and anti-tumor therapy is increasingly recognized and unravelling the mechanisms underlying this liquid metastatic microenvironment is essential to improve future efforts to eliminate peritoneal spread of tumors and optimize management of OC.

Here, we have outlined recent studies that support a key role of heterogeneous TMEs in fostering primary OC and promoting peritoneal metastases and highlighted currently available treatment attempts to combat this disease by targeting TME.

## Role of TME involved in growth, progression, prognosis and chemotherapeutic resistance of OC

TME in OC generates the only microenvironment in the peritoneal cavity referred to as “malignant ascites (MAs)” and ascites-associated OC cells are present as single-cell form or floating spherical cell clusters. The TME of OC consists of multiple cell types that support immunosuppression, along with survival, proliferation and spread of cancer cells. Non-malignant cells, including immune and stromal cells, constitute critical components of the TME. The non-malignant cellular compartment of ascites includes immune cells (e.g. T cells, monocytes, macrophages, Natural killer cells), fibroblasts, mesothelial cells and adipocytes [13, 14]. The cell-free zone of the TME is rich in extracellular matrix (ECM) proteins, growth factors, proteases, cytokines and chemokines, which contribute to the proliferation and spread of OC spheroids in the abdominal cavity [15].

**Fig. 1:**
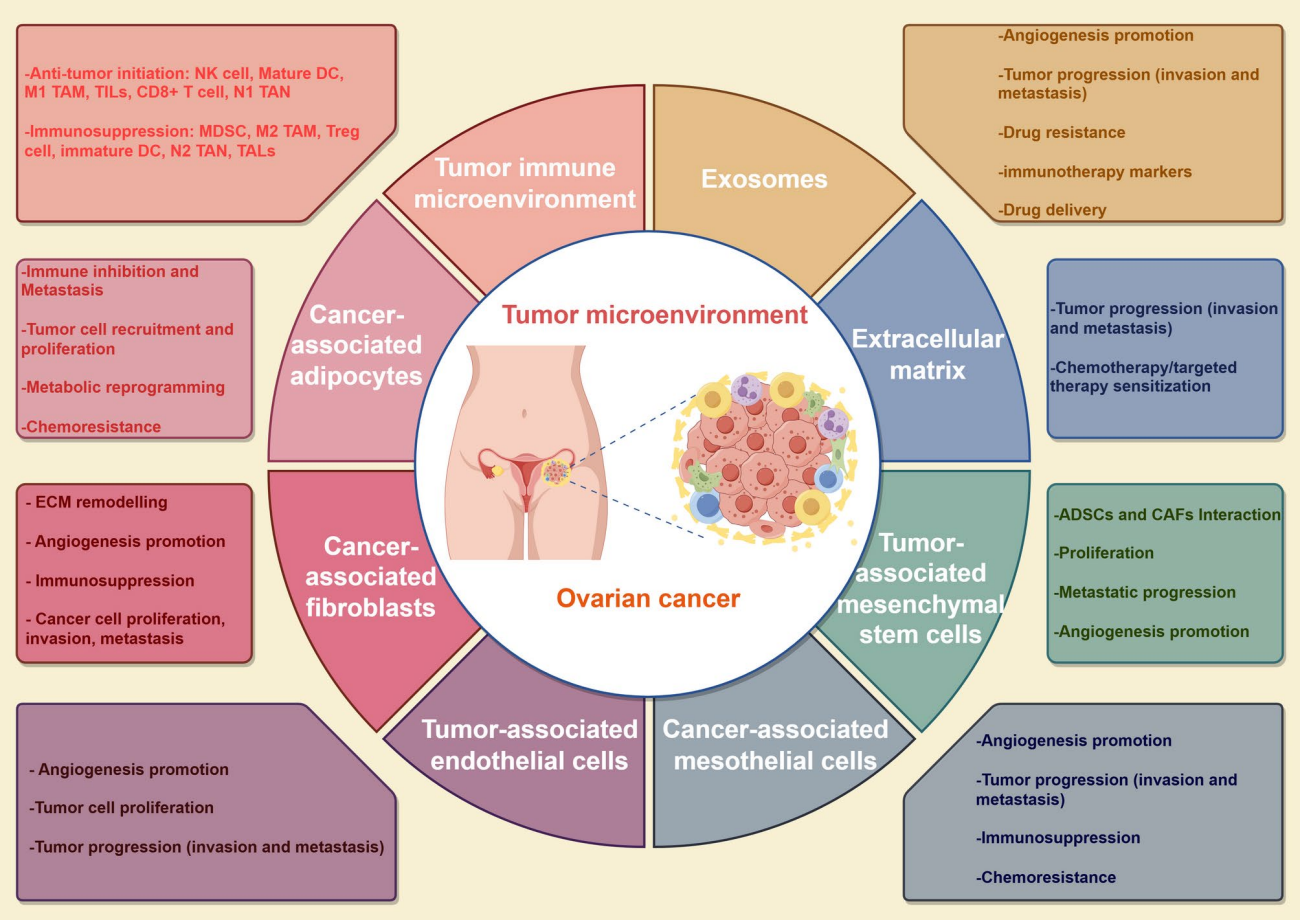
The main components of the TME and the main biological functions they perform in the OC. The TME includes both cellular and non-cellular components. Its cellular component consists of OC cells, a variety of immune cells, cancer-associated adipocytes, cancer-associated fibroblasts, tumor-associated endothelial cells, cancer-associated mesothelial cells, cancer-associated mesenchymal stem cells and exosomes. The ECM represents the non-cellular component of the TME and acts as a scaffold. Elements of the TME interact with each other through the ECM, cell-cell contacts and the release of cytokines, chemokines and extracellular vesicles. OC, ovarian cancer; TME, tumor microenvironment. ECM, Extracellular matrix

The following section synthesizes the contributions of TME components to OC growth, progression, and chemoresistance (Fig. 1). Uncovering potential interactions between cancer cells and TME may contribute to the develop novel therapeutic strategies.

### Tumor immune microenvironment (TIME)

#### Tumor-associated macrophages (TAMs)

Emerging evidence highlights the pivotal role of TAMs in mediating TME-cancer cell interactions, with significant contributions to tumor progression, invasion and metastasis [16]. TAM plasticity enables polarization into distinct phenotypes: immunosurveillant M1-like or protumorigenic M2-like macrophages. In HGSOC, M2 polarization is preferentially induced by cytokines such as colony-stimulating factor-1 (CSF-1), interleukin (IL)-6 and IL-10, which characterize the TME [138, 139]. M2-like TAM can facilitate OC progression at multiple phases of disease progression, involving tumor cell immune escape, disruption of ECM, induction of vascular regeneration and cancer-associated inflammation [140–143].

Recent studies have shown that TAMs play an important role in immune-mediated cancer control through the secretion of multiple cytokines, including IL-6, IL-10, transforming growth factor β (TGF-β), tumor necrosis factor α (TNF-α), C-C chemokine motif ligand 18 (CCL18) and CCL22 [15, 17, 18]. An example is that IL-6 released by TAMs activates the STAT3 pathway [19], which is essential for OC cell proliferation, migration, survival, and motility. Moreover, IL-6 promotes attachment, infiltration and proliferation of OC cells, potentially in part through increased expression of matrix metalloproteinases (MMPs) [20]. Additionally, the immunosuppressive chemokine CCL18 is highly abundant in OC patients and elevated levels of CCL18 facilitate tumor migration, metastasis, and are inversely associated with overall survival (OS) [21, 22]. CCL22 secreted by TAMs and OC cells attracts regulatory T cells (Tregs) to OC cell clusters, suppressing T cell immunity and enhancing tumor growth [23, 24]. In addition, TNF-α produced by TAMs promotes OC cell invasion, although the precise pathways require further clarification [25].

The primary mechanisms underlying the influence of TAMs in TME on OC also include angiogenesis, as evident from the presence of CD105-positive blood vessels in the milky white patches of the omentum, indicative of active vascular sprouting and angiogenesis [26]. It has been reported that TAM-derived MMP-9 facilitated remodeling and angiogenesis of the ECM, leading to deterioration of OC [27]. Additionally, a further finding is that TAMs that overexpress sialic acid-binding Ig-like lectin 10 interact with tumor-expressed CD24 to promote immune invasion [28]. TAMs additionally support OC cell migration and proliferation via the epidermal growth factor-epidermal growth factor receptor signaling pathway, leading to upregulation of integrins and vascular endothelial growth factor (VEGF) signaling through activation of the JNK and NF-κB pathways [25, 26, 29].

In the TME of OC, periostin (POSTN), a protein associated with hyperplasia of the osteoclasts, is highly expressed and facilitates the recruitment of macrophages via TGF-β, creating a positive feedback loop conducive to tumor development [30, 31]. TAMs also secrete B7-H4, a cytokine-reduction molecule that reduces T-cell proliferation. The presence of this molecule is related to the number of tumor-infiltrating Tregs, contributing to poor prognosis of OC via negative modulation of T-cell immunity [32]. TAMs require Zinc Finger E-Box Binding Homeobox 1 to exert a pro-carcinogenic effect by directly stimulating CCR2, and infiltration of TAMs is associated with a worse outcome in OC patients [33]. Moreover, TAMs were reported to induce the proliferative and aggressive properties of OC cells by up-regulating insulin-like growth factor-1 (IGF-1) [34].

The relevance of TAM phenotypes to the clinical prognosis of patients was examined in a meta-analysis of 794 OC patients. In this study, infiltration of CD163 + TAMs were related to adverse outcomes, while a high ratio of M1/M2 TAMs served as a predictor of improved prognosis for OS and Progression Free Survival (PFS) [35]. A separate study involving 112 advanced OC patients consistently revealed an association of a high TAM M1/M2 ratio in tumor samples with a better prognosis [18]. Another cohort study on 199 patients with high grade plasma OC demonstrated a correlation of high M2/M1 ratio with reduced PFS and poor OS [36]. Similar observations were reported by Ciucci et al. [37]. Mucin 2 (MUC2) is aberrantly expressed on OC cells and is an independent contributor to unfavorable prognosis. He et al. revealed that MUC2 expression in tumor cells negatively correlates with the M1/M2 TAM ratio, promoting cancer progression and metastasis via a TAM-dependent mechanism [38].

#### Tumor-associated neutrophils (TANs)

Recent research on the mechanisms of cancer metastasis and progression has indicated a potential pro-tumorigenic role of TANs [39–41]. In the TME incorporating an abundance of inflammatory cells and mediators, TANs are plastic and can polarize N1 phenotypes with anti-tumor activity. On the contrary, TGF-β induces N2 polarization, which induces tumorigenesis [42].

Despite the absence of specific studies directly linking TANs to the progression of OC, Lee et al. reported that OC cells release IL-8, which inhibits tumor growth. This phenomenon could potentially be associated with the recruitment of TANs [43]. In a KRAS-induced mouse model of OC, TANs were shown to exert anti-tumor effects by reducing infiltration of Myeloid-derived suppressor cells (MDSCs) and Treg cells [44]. Furthermore, neutrophils activated by LPS and IL-8 in cord blood were observed to inhibit the proliferation and invasive migration of OC cells, while simultaneously promoting apoptosis [45]. In patients, however, TANs have been reported to be recruited by chronically produced TNF-α in an IL-17-dependent manner as well as being involved in tumor promotion [46]. Similarly, TANs may be activated by mitochondrial DNA present in ascitic fluid, thereby impairing anti-tumor immunity and resulting in reduced PFS in patients with OC [47]. Klink et al. [48] revealed that direct interactions between TANs and OC cells derived from OC patients resulted in increased production of reactive oxygen species, enhanced adhesion function, and upregulated expression of CD11b and CD18, in comparison to TANs obtained from healthy female volunteers. More recently, high levels of TANs have been reported to be associated with poor prognosis and immune tolerance in OC. They indicated that TANs modulate the cytotoxic efficacy of CD8 + T cells in part via the Jagged2 (JAG2) pathway. Furthermore, JAG2-positive TANs are intricately linked to the IL-8-mediated immune evasion microenvironment and could serve as a therapeutic target to boost anti-tumor immunity [49]. Elucidation of the modification profile of TANs and the underlying molecular pathways should facilitate the identification of potential actionable markers for OC.

TANs can be an indirect indicator of the state of inflammation. Numerous studies have revealed that an increased NLR serves as a significant prognostic indicator associated with increased incidence of recurrence in multiple cancer types [50–53]. The potential role of NLR in OC has been extensively researched. According to the findings, increased levels of preoperative NLR is an indicator of low patient survival and platinum resistance [54–59]. In addition, a high preoperative NLR rate was associated with a high morbidity rate and poor OS at 30 days postoperatively [60]. Similarly, Nakamura et al. [61] confirmed an association between elevated NLR values and increased mortality within 100 days of chemotherapy in OC patients. Additionally, preoperative NLR combined with CA125 has been proven to improve early diagnostic accuracy [62]. The aggregated findings suggested a potential immunomodulatory function of TANs in OC.

#### MDSCs

MDSCs represent an intrinsically heterogeneous cell group, expanding upon malignant transformation, inflammation, and infection [63, 64]. MDSCs are pathologically conditioned to induce growth and perform immunosuppressive effects through modulating evasion of anti-tumor T-cell immune responses [65–67]. In the TME of OC patients, MDSC levels are elevated, with a correlation between the abundance of MDSCs and a reduced survival rate [68–70]. An earlier study by Montalban and co-workers found elevated levels of IL-6 and IL-10 as well as MDSC concentrations associated with poor prognosis in OC patients [71]. Similar conclusions were obtained from another study, whereby IL-6 and IL-10 play an indirect role in promoting the recruitment of MDSCs in peripheral blood and ascites of OC patients via STAT3 activation, which was linked to poor prognosis [70]. VEGF and adenosine produced by OC cells additionally facilitate MDSC recruitment, with consequent suppression of local immunity [72]. In patients, MDSCs could further acquire tolerance through DNA methyltransferase 3 A- and prostaglandin E2 (PGE2)-dependent hypermethylation, which is necessary for cells to develop immunosuppressive potential, providing a novel avenue for therapeutic interventions in OC [73].

Notably, increased levels of MDSCs in tumors were negatively correlated with CD8 + tumor-infiltrating lymphocytes (TILs) and were significantly correlated with tumor progression and survival in advanced-stage OC patients [74]. Evidence also suggested that MDSC infiltration was linked to shorter OS and higher serum levels of C-X-C Motif Chemokine Receptor 2 (CXCR2), regulated by the transcription factor Snail involved in epithelial-mesenchymal transition [75]. Importantly, the data obtained by Taki and colleagues indicate that MDSCs enhance “stemness”, potentially linked to resistance to classical anticancer therapies [76].

MDSCs have recently been implicated in promoting tumor progression and impairing T-cell function in a preclinical model of OC [74]. Interestingly, Li et al. [72] administration of metformin to diabetic OC patients resulted in a reduction of circulating MDSCs, an increase of circulating CD8 + T cells, and an extension of survival. This finding support immunotherapeutic strategies that specifically target MDSCs to enhance anti-tumor responses. For example, the suppression of MDSCs may be blocked by suppressing CD39 and CD73 expression with metformin, which is a drug used in the treatment of type 2 diabetes mellitus. By enhancing the anti-tumor T-cell reactions in TIME that are suppressed by MDSCs, this blockade may promote clinical benefit in HGSOC [77]. The key function of MDSCs in regulating T-cell responses and tumor progression support their utility as potent biomarkers for patient selection in OC and as novel targets for potential therapeutic interventions.

Based on the density, morphology, and phenotype, MDSCs fall mainly into two subsets: polymorphonuclear (PMN)-MDSCs and monocytic (M)-MDSCs [63]. PMN-MDSCs and M-MDSCs have different functions and biological characteristics under various pathological conditions. PMN-MDSC (not M-MDSC) has been reported to be the major population that downregulates T cell immune activity [78]. A growing number of studies have shown that PMN-MDSCs exert their immunosuppressive effects mainly by enhancing the expression of arginase 1 (ARG1), TGF-β, IL-10 and indoleamine 2,3-dioxygenase (IDO) [68, 79, 80]. One study confirmed that PMN-MDSCs are closely associated with poor outcomes in OC patients. They found that deletion of the ANKRD22 gene increased the expression of immunosuppressive molecules (such as Arg1, iNOS, IDO, and PD-L1) in PMN-MDSCs, and also increased the chemotactic and immunosuppressive activity of PMN-MDSCs in local tumors, indirectly promoting the growth of OC cells by inducing the formation of an immunosuppressive microenvironment [81].

Recently, a study performed a comprehensive analysis of each MDSC subset and immunosuppressive factors in peripheral blood, ascites, and tumor tissue samples from OC [68]. The results showed that the levels of M-MDSCs in the peripheral blood/ascites/tumor tissue of OC patients were significantly higher than those in healthy donors (HD); the frequency of PMN-MDSCs in tumor tissue was significantly higher than that in peripheral blood/ascites and HD. At the same time, combined with clinical data, it was found that the high abundance of tumor-infiltrating M-MDSCs was associated with an increase in the tumor stage and grade of OC. In addition, analysis of the immunosuppressive pattern exhibited that compared with HD, OC patients had a significant increase in ARG/IDO/IL-10-expressing M- and PMN-MDSCs in the blood, and this accumulation was positively correlated with plasma levels of TGF-β and ARG1 [68].

In addition, a research team confirmed that the ratio of M-MDSC/DCs in the blood is an independent predictor of OC survival [69]. They showed that the number of M-MDSCs in the peripheral blood and ascites of OC patients was significantly increased compared to HD and negatively correlated with the patients’ recurrence-free survival. Interestingly, they found that ascites from OC patients can easily induce M-MDSCs, which is mainly dependent on the activation of STAT3 pathway, thereby upregulating the expression of ARG1 and inducible nitric oxide synthase in induced M-MDSCs. These MDSCs perform immunosuppressive activities through these enzymes. Therefore, improving anti-tumor efficacy by locally targeting MDSCs may be a new therapeutic option [70].

#### Dendritic cells (DCs)

DCs serve as a critical interface between innate and adaptive immunity by presenting antigens to antigen-presenting cells (APCs). Mature dendritic cells are required for the initiation and maintenance of T-cell-dependent anti-tumor immunity [82]. DCs are classified into two main subpopulations based on functional and phenotypic characteristics: conventional DCs (cDCs), which specialize in antigen presentation, and plasmacytoid DCs (pDCs), which produce interferon-alpha (IFN-α) following antigen stimulation [83, 84]. Each DC subpopulation mediates the immune system through distinct mechanisms. In an inactive state, these cells roam the body in an immature form and function in the detection of phagocytic pathogens. Following infiltration, DC function is adversely affected by the TME of OC, leading to impairment of anti-tumor immune responses mediated by T cells [85–87].

According to recent studies, DCs infiltrating OC tumors can affect patient prognosis depending on the subpopulation. TMEs with a high density of pDCs are often immunosuppressive and have poorer clinical outcomes. OC cells can repel DCs with angiogenesis-inhibiting properties and attract pDCs that induce angiogenesis through secretion of TNF-α and IL-8 [88]. In a cohort study, aggregation of CD4 + BDCA2 + CD123 + pDCs within the TME was associated with early recurrence [89]. In addition, pDCs are reported to be an important part of Treg cell-mediated immunosuppression, resulting in progression of OC [90].

In the study conducted by Wei et al. [91], it was demonstrated that TApDCs induce immunosuppressive CD8 + T lymphocytes in OCs. Phenotypic and functional distinctions between TApDCs and pDCs in advanced OC have been identified, corroborating the hypothesis that pDCs exhibit pro-inflammatory characteristics, whereas TApDCs demonstrate pronounced immunosuppressive properties and are linked to early recurrence and unfavorable prognosis [89, 92]. In OC cells, a subset of TApDCs exhibit endothelial and pericyte characteristics and are hypothesized to contribute to tumor vasculoprotection. Previous studies have demonstrated that depleting these cells causes vascular apoptosis, tumor necrosis, and enhances chemotherapy and anti-tumor immune responses [93]. Furthermore, OC cells have the capacity to undermine the functionality of DCs by disrupting their activation, antigen presentation, differentiation, and recruitment processes, thereby facilitating immune evasion. In the presence of activated endoplasmic reticulum stress responsive element XBP1, and TApDCs under conditions of stress could impair anti-tumor immunity, thereby driving OC progression [94].

The presence of mature DC-LAMP + dendritic cells in conjunction with CD20 + B-cell infiltration is associated with extended OS in chemotherapy-naïve patients with HGSOC, thereby underscoring the potential of dendritic cells as prognostic biomarkers [95]. In OC patients, bone marrow DCs in draining lymph nodes upregulated programmed death-ligand 1 (PD-L1) receptor expression and therefore failed to initiate and maintain T cell activation [96, 97]. Furthermore, it has been demonstrated that PGE2 and cyclooxygenase 2 (COX2) induce the differentiation of CD1a + DCs into CD14 + CD33 + CD34 + MDSCs, thereby contributing to immunosuppressive mechanisms within the TME [98].

Immunotherapy based on DCs may be a useful treatment option for OC. Several recent studies have focused on designing DC vaccines for activating responses of tumor antigen-specific Th17 T cells, which, in combination with adjuvant therapies, eliminate immunosuppressive mechanisms in the TME, offering potential clinical benefits [99–101]. A further advantage of whole tumor lysates is that they provide a rich source of antigen for DC therapy due to an abundance of relevant immunogenic epitopes that aid in preventing tumor escape. DC vaccines incorporating hypochlorite-oxidized tumor lysate illustrated efficacy in augmenting T-cell-mediated antitumor immunity and in prolonging PFS in patients with recurrent OC [102].

#### Natural killer cells (NK cells)

NK cells are important innate immune lymphocytes that secrete a range of pro-inflammatory cytokines and chemokines upon activation, including IFN-γ, TNF-α, IL-6, CSF and CCL5 [103, 104]. Research has revealed that TME-induced aberrant molecules regulate the antitumor response of NK cells. Notably, one study identified and characterized a subpopulation of mature human NK cells exhibiting overexpression of PD-1 within the ascites of OC patients [105, 106]. PD-1 + NK cells are less responsive to exogenous cytokines for proliferation and exhibit diminished anti-tumor activity [106]. OC cells induce T-cell dysfunction via ULBP2 expression, a mechanism shared by other cancers [107].

An earlier study suggests that increased lysis of cancer targets by CD56^bright^ NK cells is not related to elevated cytokine production. CD16 + NK cells are implicated in cytotoxic responses; however, their prevalence is markedly diminished in HGSOC ascites [108]. In addition, the existence of NK cells in-stage OC exudate is predictive of lower OS in patients [109]. However, NK cells can also perform positive anti-tumor functions along with effector CD8 + T cells [110]. CD57 + and CD103 + intratumoral NK cells positively correlate with improved survival among patients with HGSOC, similar to CD8 + TILs [110, 111].

Despite their intrinsic capacity to identify transformed cells, NK cells are susceptible to immunosuppression mediated by TMEs and may experience dysfunction induced by TMEs. For example, TAMs within the TME of OC patients enhance the production of migration inhibitory factors by down-regulating the NK activation receptor NKG2D, resulting in immune evasion [112]. In addition, NK cells form subpopulations of pro-carcinogenic and hypofunctional cells in the TME of HGSOC via retrograde phagocytosis of CD9 and inhibition of receptor upregulation [113]. NK cell activity within ascitic fluid is suppressed by elevated concentrations of soluble B7-H6, a ligand for the NKp30 receptor. The increased expression of soluble B7-H6 correlates with diminished levels of NKp30 and compromised functionality of tumor-associated NK cells [114]. It is consistent with this observation that reduced expression of B7-H6 in patients is associated with improved OS, as well as decreased tumor metastasis and progression [115].

CD57 + NK cells have a more favorable prognostic impact in patients with HGSOC tumor infiltration. Henriksen et al. [111] reported that compared to patients exhibiting low levels of CD56 + NK cells, those with a high proportion of CD57 + NK cells indicated a significantly prolonged OS. Similarly, IL-15 augmented the immune response in OC patients by increasing the proportion of CD56 + NK cells in their ascitic fluid, indicating a promising avenue for the development of novel immunotherapies [116].

In recent years, NK cells have garnered significant attention as potential targets for immunotherapeutic interventions [117–119]. The in vitro activation, expansion, and genetic modification of NK cells have the potential to mitigate drug resistance and enhance their anti-tumor efficacy. In a study conducted by Nham et al., artificial APC-based in vitro expansion techniques were employed to generate cytotoxically enhanced NK cells, which were utilized within an autoimmune therapy model [120]. NK cells isolated by this research group from Mas of OC patients exhibited enhanced surface expression of activation receptors, which are responsible for the production of anti-tumor cytokines and the direct cytotoxicity against OC cells. Based on these results, we can consider MAs of OC patients as a potential cytotoxic NK cell source, thereby offering a potential immunotherapeutic target for the second-line treatment of OC [120].

#### Adaptive immune cell populations

Within the context of the adaptive immune system, B and T lymphocytes are prevalent, with T lymphocytes being particularly abundant in OC tissues and TME [121]. Given the pivotal role of T lymphocytes in tumor immunosurveillance, this section will concentrate on the various subpopulations of these cells.

T cells present in primary or metastatic tumors are designated as TILs, while those found in ascitic fluid are referred to as tumor-associated lymphocytes (TALs) [122]. There has been considerable research on TILs and their potential utility as predictive biomarkers in OC patients over the past two decades. There is a positive correlation between TILs and a favorable outcome, because these cells can control tumor growth by activating anti-tumor immune responses [123]. Despite TILs being an independent prognostic factor, the equilibrium among various TIL subpopulations significantly influences the immune response. Numerous studies have examined the composition of TILs across various stages of OC. Among infiltrating T cells, CD8 + T cells are associated with improved outcomes, while CD4 + T cells expressing Forkhead box P3 (FOXP3) appear to counteract these benefits [108, 124–126]. A meta-analysis encompassing 10 studies and involving 1,815 patients with OC elucidated the prognostic significance of intraepithelial CD8 + TILs in OC specimens, independent of tumor grade, stage, or histological subtype. Furthermore, the absence of TILs was significantly correlated with reduced survival rates in OC patients [127]. Several studies have confirmed an association of improved disease-specific survival with the occurrence of intraepithelial CD8 + TILs in primary or metastatic lesions of OC patients [128–130].

A study involving 186 patients with advanced OC revealed that the presence of CD3 + TILs was associated with a 5-year OS rate of 38.0%, in contrast to a significantly lower rate of 4.5% observed in patients lacking CD3 + TILs [131]. CD3 + TILs were found to improve 5-year OS to more than 70% following surgery and platinum-based chemotherapy, compared to only 11% in patients with tumors devoid of TILs [127]. Furthermore, research has demonstrated that an elevated CD8+/CD4 + TAL ratio is associated with a favorable prognosis, whereas a higher CD4+/CD8 + ratio is indicative of a less favorable outcome [132]. The reduction in mortality among OC patients was linked to the expression of COX-1 and COX-2, which exhibited a negative correlation with the presence of intraepithelial CD8 + TILs [133]. In addition, the absence of TIL in tumors was correlated with elevated levels of VEGF, a regulatory factor of angiogenesis in TIME, which is linked to early recurrence and reduced survival rates in OC patients [134]. Moreover, CD3+, CD4+, CD8+, and CD103 + TILs have been associated with a longer OS and PFS in another meta-analysis of 19 studies including 6004 patients with HGSOC [135].

Notably, Tregs are integral to the modulation and regulation of the immune response [136]. A critical source of immunosuppression in the TME is CD4 + Tregs, which are significantly enriched in tumors of cancer patients [137]. The presence of Tregs in TME is correlated with the progression of advanced-stage disease. Notably, Tregs identified within TALs in ascites of OC patients exhibit a phenotype indicative of heightened activation relative to circulating Tregs, thereby implicating the TME in the modulation of Treg activity [23, 138]. Tregs are recognized as expressing CD4, CD25 and FOXP3. In solid tumors, CD4 + CD25 + FOXP3 + Tregs mediate immunosuppression via a mechanism dependent on the COX2/PGE2 pathway [139, 140]. Hypoxia-induced CCL28 and CCL22 recruit Treg cells in tumor and ascites, thereby promoting immune privilege, in turn, sustaining cancer cell growth. A high risk of death is also associated with the accumulation of FOXP3 + Treg cells in OC patients [23, 141].

The TME in OC favors the induction and differentiation of Tregs through multiple pathways and the presence of Tregs is linked with poor prognosis [142]. Tregs have been documented in numerous studies to be present within TALs in OCs [126, 138, 143] and an inverse association of their accumulation with patient survival [23, 144]. According to the study conducted by Peng et al. [145], Tregs are present in various populations, and their potential clinical applications in OC have been systematically reviewed. It is unequivocal that OC cells influence the phenotype of immune cells, as evidenced by the research conducted by Alvero et al. [146]. Their study identified two distinct subpopulations of OC cells, characterized by divergent cytokine profiles: differentiated cancer cells and cancer stem cells. Treg production is increased in differentiated cancer cells, creating a tolerogenic microenvironment that suppresses the immune response, which is linked to poor survival. Given that Tregs are significantly modulated by microenvironmental factors, strategies to reprogram these cells may present an effective alternative therapeutic approach for OC.

The infiltration of tumors by CD8 + T cells serve as an indicator of immune recognition and is predictive of enhanced survival outcomes in patients with OC. The regulatory role of CD8 + T cells in OC TME is crucial as these cells can remove tumor cells by secreting granzyme B, TNF and IFN-γ [147]. However, CD8 + T cells commonly tend to be dysfunctional in the immunosuppressive microenvironment in most cases. An earlier study exhibited that only 10% of intratumorally CD8 + T cells could detect autologous OC cells and tumor-reactive T-cell receptors were absent from half of the patient samples [147]. This phenomenon may be partially attributed to the depletion of CD8 + T cells. Prolonged exposure to antigens within the tumor microenvironment results in the functional impairment of T cells, characterized by a loss of effector functions, upregulation of inhibitory receptors such as PD-1, and a diminished capacity for memory recall. The OC microenvironment compromises the anti-tumor efficacy of CD8 + T cells through the inhibition of various signaling pathways [148, 149]. In addition, inhibitory signals from ligands on APCs, tumor cells, and TILs can be targeted by advanced immunotherapy to potentially enhance prognosis [150, 151].

Several cytokines and chemokines influence OC prognosis: IL-2, IL-5, IL-7, and CCL5 are linked to better outcomes, whereas IL-6, IL-8, IL-10, TGF-β, CCL2, and VEGF are associated with worse outcomes [132, 133, 152–156]. These potential candidate biomarkers for OC could offer valuable insights into disease development and promising avenues for tumor-targeted therapy.

**Fig. 2:**
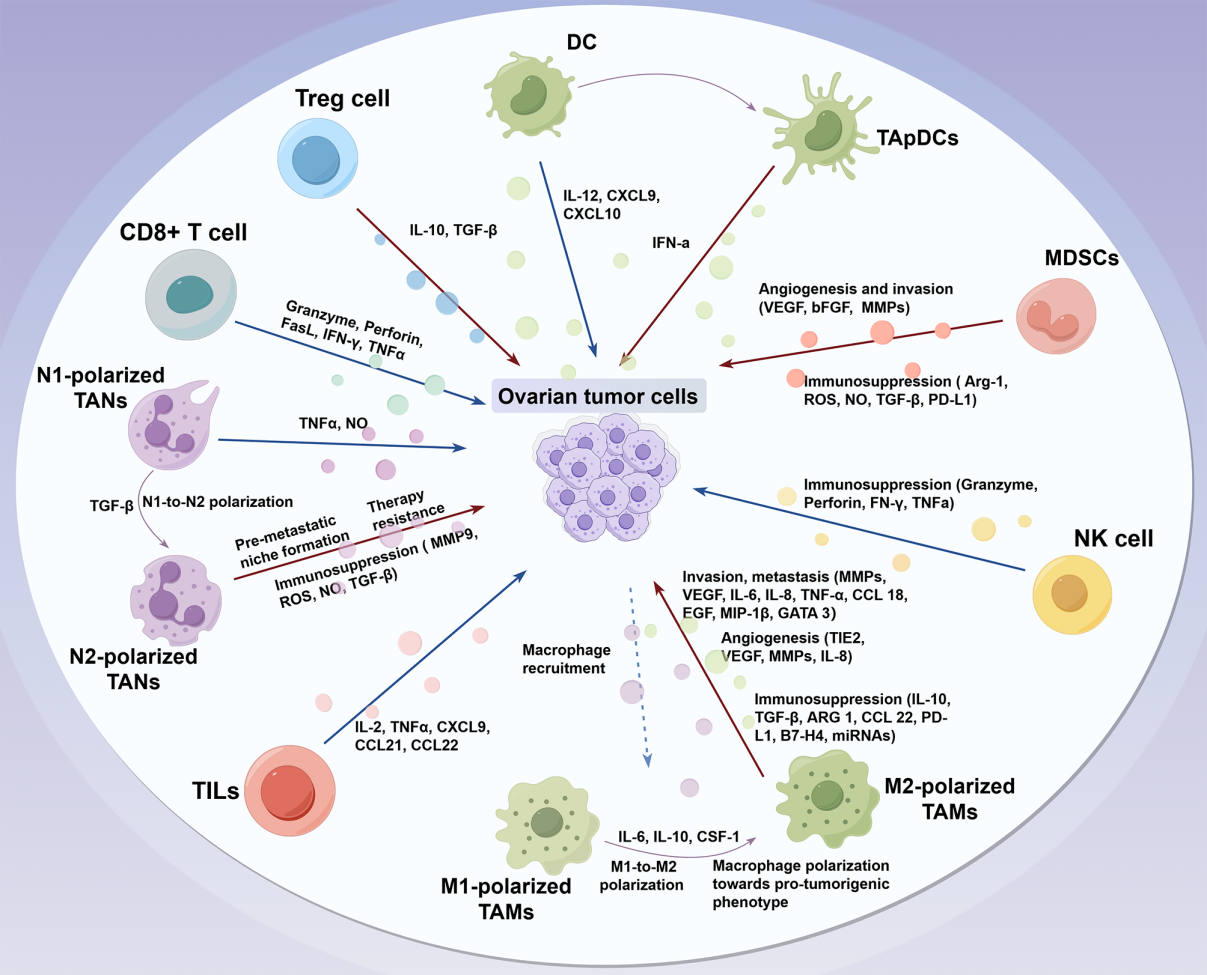
Schematic representation of the TIME. Multiple immune cell subpopulations are present in the TIME, which play an important role in OC development, progression and metastasis. Red arrows represent the functions of pro- tumoral cells, including Treg cells, MDSCs, TAMs and immature DC cells, which promote tumor escape. Blue arrows represent the functions of anti-tumoral cells, such as CD8 T cells, mature DC cells, NK cells and TAN cells, which contribute to tumor killing. OC, ovarian cancer; TIME, tumor immune microenvironment; MDSCs, Myeloid-derived suppressor cells; TAMs, Tumor-associated macrophages; DC cells, Dendritic cells; NK cells, Natural killer cells; TAN, Tumor-associated neutrophil

Figure 2 summarizes TIME components and their roles in OC progression. By analyzing the distinct classes and subclasses of the TIME present in patients’ tumors, it is feasible to enhance the predictive accuracy and guidance of immunotherapy responsiveness. This approach may consequently facilitate the identification of novel therapeutic targets.While immune cells play a pivotal role in modulating the TME, the adipose microenvironment further contributes to ovarian cancer progression by providing metabolic support and facilitating chemoresistance. The following section will explore how adipocyte-derived factors interact with tumor cells and immune components.

### Adipose microenvironment

Research has demonstrated that the omentum, which is rich in adipose tissue and serves as the primary site for OC metastasis as well as the most frequent location for residual and recurrent disease, is implicated in the progression of OC [157–160]. OC cells disseminated within the abdominal cavity exhibit a pronounced propensity for metastasizing to the omentum, the adipose tissue associated with abdominal organs, and the adipose tissue enveloping the mesentery, liver, and kidneys [161]. Cancer-associated adipocytes (CAAs) are an integral part of the TME. These multifaceted and evolving cells play multiple roles in construction of the TME [162].

CAAs can induce OC cell homing by secreting adipokines and inflammatory factors, including leptin (LEP), adiponectin, IL-6, IL-8, MMP-11 and CCL5. Nieman and colleagues demonstrated that CAAs provided fatty acids via fatty acid-binding protein 4 (FABP4), which promoted the growth of OCs. Additionally, the omental adipose cells co-cultured with OC SKOV3 cells stimulate OC cell homing, migration and invasion, both in vitro and in animal models [163]. Cytokines secreted by adipocytes, including IL-8 and IL-6, have been reported to promptly activate the AKT and ERK survival pathways in OC and to upregulate various genes associated with OC survival [20, 164].

Crosstalk between cancer cells and adipocytes supports the rapid proliferation and invasion of OC [165–167]. Salt-induced kinase 2 is increased in adipose tissue-related OC cells, enhancing fatty acid oxidation, whereas the PI3K/Akt pathway drives cancer cell growth and survival [168]. Adipocyte-derived signals, including inflammatory mediators, can augment lipid uptake in OC cells via the STAT3/FABP4 signaling pathway, thereby promoting increased cellular proliferation both in vitro and in vivo [169]. Deleting SPARC, however, inhibits adipocyte differentiation, OC migration and homing to lipid-rich niches, and metabolic reprogramming of OC cells [170].

Studies indicated that LEP (an adipokine secreted by adipose tissue) promotes OC invasion, proliferation, and chemoresistance via cell cycle activation and anti-apoptotic pathways. Clinically, LEP overexpression correlates with advanced tumor stage and recurrence [171–173]. Additionally, LEP in obese patients may play a role in the maintenance and survival of dormant cancer cells that persist following surgical resection, particularly in locations such as ascites or the peritoneal cavity, which, in turn, increases the risk of disease recurrence [174]. There is evidence that LEP contributes directly to chemoresistance in OC cells [175, 176]. LEP expression is linked to poor outcomes in platinum-treated patients and decreased chemosensitivity of OC cells to platinum, paclitaxel, and docetaxel [177]. LEP also activates AKT and ERK survival pathways in OC cells, crucial for drug resistance [178]. The collective results highlight the potential of LEP neutralization as an innovative approach to augment OC therapy.

Studies have demonstrated that the adipose microenvironment contributes to chemotherapeutic resistance in OC and response to chemotherapy in adipose-associated metastatic disease is correlated with survival [160, 179]. In addition, a study of 161 stage III-IV HGSOC patients revealed significant prognostic value of the chemotherapy response score for omental disease in relation to both OS and PFS [179]. Similarly, significantly poorer prognosis of patients with stage III-IV OC with omental metastases due to increased chemotherapy resistance has been reported [180]. The anti-apoptotic protein Bclxl mediates chemoresistance induced by adipocytes. It is significantly upregulated in chemoresistant CD44+/MyD88 + OC stem cells compared to sensitive CD44-/MyD88- cells. In vitro, a factor secreted by adipocytes induces Bclxl expression in CD44-/MyD88- cells, leading to carboplatin resistance [181].

Adipocytes have also been shown to indirectly promote chemotherapeutic resistance in OC. An earlier study revealed significant alterations in FABP4 in co-cultures of SKOV3ip1 human OC cells with human omental biopsy tissue. Moreover, inhibition of FABP4 induced resistance to carboplatin in human OC cell lines [182]. Elevated levels of FABP4 have been demonstrated to correlate with higher recurrence rates following surgical intervention for HGSOC, supporting the utility of FABP4 as a biomarker of prognosis for OC recurrence [183]. Adipocytes indirectly promote chemoresistance by remodeling the ECM, notably through collagen VI overexpression. They secrete large amounts of collagen VI when in close contact with cancer cells [184]. OC cells adhering to collagen VI show increased survival and resistance upon exposure to cisplatin, which could be achieved via upregulation of metallothionein [185]. A report by Yang et al. [186] demonstrated that arachidonic acid from adipocytes increased OC cell resistance to chemotherapy by activating the Akt pathway.

In summary, the adipose microenvironment is the primary site for OC metastasis and recurrent disease. It influences OC growth, including cell proliferation, migration, chemoresistance, and metabolic adaptation. These insights offer potential for new therapies, with targeting adipocyte-derived factors as a promising strategy to combat chemoresistance.

### Cancer-associated fibroblasts (CAFs)

In addition to immune cells and the adipose microenvironment, fibroblasts in the stroma further shape the pre-metastatic niche of OC by remodeling the ECM and secreting cytokines. Fibroblasts, which constitute a fundamental component of the stromal tissue, are induced by various proliferative signals to differentiate into activated fibroblasts, commonly referred to as CAFs [187], with a faster proliferative capacity and higher metabolic status compared to their normal counterparts. These cells release cytokines that, through paracrine signaling in the TME, directly stimulate the proliferation, differentiation, invasion, and metastasis of nearby tumor cells, while also indirectly modulating the immune system and influencing tumor metabolism [188]. Better understanding of CAF mechanisms in OC could lead to effective CAF-targeted therapies.

Notably, TGF-β is abundantly present in OCs and is essential for activating CAFs, promotion of tumor pathogenesis and avoidance of immunomodulation, ultimately resulting in the formation of a favorable TME [189, 190]. Collagen triple helix repeat-containing-1 (CTHRC1), collagen type XI alpha 1 (COL11A1), POSTN, and versican (VCAN) are genes associated with TGF-signaling pathways in CAFs, which are critical for the interaction between fibroblasts and OC cells. The encoded proteins are involved in CAF activation and tumor pathogenesis. The upregulation of CTHRC1 facilitates tumor invasion and migration via the epidermal growth factor (EGF) receptor/ERK1/2/AKT signaling pathway. Additionally, it plays a role in modulating the immune response and promoting angiogenesis, thereby contributing to tumor progression [191]. COL11A1, a mediator of stromal-cancer cell crosstalk, is upregulated and activates CAFs via modulation of TGF-β3 through the NF-κB/IGFBP2 axis [192]. By activating the TGF1/MMP3 axis, COL11A1 contributes to tumor invasiveness and poor prognosis [192]. POSTN enhances M2 TAMs and CAFs through integrin-mediated TGF-β2 and NF-κB signaling, thereby promoting growth and metastasis of OC [193]. Furthermore, upregulation of the TGF-β/TGF-βR/Smad pathway in CAFs is shown to induce overexpression of genes in the form of VCAN and subsequent targets of gene secretion, which are involved in migration and invasion via CD4 binding and activated by NF-κB and JNK signaling pathways. These support the possibility that OC cells further facilitate the pro-inflammatory TME and tumor evolution [194]. HOXA9 is a Müllerian model gene that exhibits elevated expression levels in OC cells that activates the transcription of TGF-β2. Activated TGF-β2 induces expression of VEGF-A, IL-6 and CXCL12 in CAFs, further creating a microenvironment favorable for OC progression [195].

Other studies likewise present evidence of potential molecular mechanisms implicated in the crosstalk between cancer cells and CAFs that facilitate tumor invasion. For example, CAFs attenuate immune responses via miR141/200a-mediated regulation of CAF-derived CXCL12 expression. This chemokine enhances the infiltration of immunosuppressive CD25 + FOXP3 + T lymphocytes within the HGSOC microenvironment, thereby promoting tumor progression [196]. Research indicates that Hedgehog (Hh) signaling regulates the stromal microenvironment, fostering cancer metastasis. In a mouse model, blocking Hh signaling in CAFs lowered VEGF-C levels, reducing tumor growth and lymphangiogenesis. These findings highlight the role of CAFs in cancer lymphangiogenesis via the Hh/VEGF-C pathway and suggest Hh inhibitors could be beneficial in OC treatment [197]. Moreover, CAFs promote tumor growth, spread, and invasion by releasing significant levels of mitogenic factors, including fibroblast growth factor-1 (FGF-1) and hepatocyte growth factor (HGF) [198–200]. According to a recent study, IL-8 secreted by CAFs and OC cells promotes stemness through activation of Notch3-mediated signaling pathways. This finding suggested a potential avenue for the development of innovative therapeutic strategies targeting OC [201].

Additionally, CAFs are widely known to stimulate immunosuppression and angiogenesis. Both CAFs and OC cells release chalcogenide intracellular channel protein 3, a glutathione-dependent oxidoreductase that enhances angiogenesis and cancer cell invasiveness via transglutaminase-2-dependent invasion, in vivo and in three-dimensional cell cultures [202]. CAFs additionally express Dickkopf-3 (DKK3), which is associated with the invasive profile of OCs. DKK3 connects YAP/TAZ and HSF1 signaling pathways, inducing a pro-tumorigenic phenotype in CAFs [203]. Moreover, CAF-secreted CXCL14 drives the expression of Long Noncoding RNA LINC00092 in OC cells, thereby facilitating OC growth and invasion. LINC0009 interacts with 6-phosphofructo-2-kinase/fructose-2,6-bisphosphatase 2 to induce a glycolytic phenotype in OC cells [204]. CAFs promote disease progression in epithelial cancer cells by boosting autophagy through the release of pro-inflammatory cytokines, autophagy-derived substrates, and metabolites [205].

Several studies have identified CAFs as biomarkers of poor prognosis in OC. A positive correlation between CAF-derived flavin-containing monooxygenase 2 (FMO2) and CD163 + cell infiltration in OC tissues has been reported. The co-expression of FMO2 and infiltration of CD163 + cells within the tumor stroma serves as a prognostic indicator of reduced OS [206]. In addition, a study on CAFs in HGSOC by Givel and workers reported an association of CXCL12β expression and infiltration of CAF-S1 (a subtype of CAFs) with poor prognosis [196]. A recent study found two CAF subtypes: tumor-promoting CAF_c1 and myofibroblast-like CAF_c2. Patients with higher CAF_c1 expression had worse prognosis and were more resistant to immunotherapy [207]. These findings offer insights into therapeutic strategies involving CAF regulation, indicating that patient selection should take into account CAF status.

Additionally, CAFs can exert compressive forces on microvessels and form physical barriers, thereby impeding the delivery of chemotherapeutic agents and contributing to the development of chemoresistance [208]. For instance, cysteine and glutathione synthesized by CAFs inhibit the accumulation of platinum-based chemotherapeutic agents in OC cells [209]. In some cases, CAF markers, such as CD44, or CAF isoforms, like CD10 + GPR77 + CAFs, help to maintain the stemness of cancer cells, thus promoting chemoresistance [210, 211]. Furthermore, CAF-driven upregulation of lipoma-preferred partner expression is reported to increase microvascular endothelial adhesion and regulate stress fiber formation, thereby inducing chemoresistance [212]. Another investigation revealed that miR-21 in exosomes metastasizing to neighboring CAFs inhibits OC apoptosis, leading to failure to respond to chemotherapy [213]. Similarly, The exosomes derived from CAF induce cisplatin resistance in OC by downregulating CDKN1A [214]. These results lend credence to another potential strategy for preventing tumorigenesis and drug resistance.

Other CAF-related signaling pathways are associated with chemoresistance in OC. For example, CAF-associated paracrine signaling leads to poorer prognosis and potential chemoresistance [215]. Additional mechanisms by which CAFs may promote chemoresistance of OC include direct inhibition of X-linked inhibitor of apoptosis protein and regulation of the PI3K/AKT pathway [216]. A recent study found that CAFs and tumor cells jointly activate the JAK/STAT pathway, forming the ascites system that enhances tumor growth and induces resistance to therapy [217]. Moreover, CAFs activate the Wnt/β-catenin pathway in OC cells via the CXCL12/CXCR4 axis, in turn, inducing EMT and cisplatin resistance [218]. Notably, CD8 + T cells modify cystine and glutathione metabolism in CAFs via the JAK/STAT1 pathway, reducing CAF-induced resistance to platinum chemotherapy [209].

Overall, CAFs represent a key component of the TME that contribute significantly to growth, progression and metastasis, and the treatment resistance of OC. Further research on the development of early diagnostic tools and therapeutic approaches specifically targeting CAFs is necessary.

### Tumor-associated endothelial cells (TAECs)

Angiogenesis, the formation of new capillaries from existing blood vessels, is vital for the progression and peritoneal spread of OC [219]. In OC, up to 70% of cases express VEGF. Several studies have explored the differential expression of the VEGF gene in tumor specimens relative to benign ovarian tissues [220, 221]. VEGF levels are additionally significantly elevated in OC-induced MAs, with prognostic significance [222–224]. Similarly, another study also showed that elevated VEGF levels correlate with ascites formation and tumor burden [225].

The clinical diagnosis and prognostic assessment in patients with OC are presently facilitated by the utilization of serum VEGF biomarkers [226]. Earlier studies have identified elevated preoperative VEGF levels as an independent risk factor for disease-related mortality [227]. VEGF-C in ascites of OC patients is associated with FIGO stage, tumor grade and lymph node metastasis stage. Furthermore, VEGF-C concentration is an independent predictor of reduced OS [228]. Another survival analysis further confirmed a strong correlation of elevated VEGFR1 expression in OC with reduced OS and PFS [229]. VEGF gene polymorphisms have been established as independent poor prognostic indicators of OS [230].

A number of other angiogenic targets are currently under investigation. Notably, the upregulation of Enhancer of Zeste Homolog 2 (EZH2) expression has been demonstrated to facilitate angiogenesis. EZH2 silencing in tumor-associated endothelial cells led to inhibition of vasohibin1 reactivation-mediated angiogenesis and OC growth in a previous study by Lu et al. [231], supporting the potential of targeting EZH2 as an effective therapeutic approach. Migration inhibitory factor (MIF) is overexpressed in OC cell lines and MAs of OC. Depletion of this chemokine leads to a reduction in tumor vascularity and the proportion of endothelial cells in ascites. Angiogenesis may be promoted by MIF as a result of stimulation of VEGF and inflammatory cytokines, such as TNF-α and IL-6 [232]. In addition, angiogenesis and immune tolerance that facilitate maintenance of OC cell survival are determined by hypoxia-driven expression of CCL28 and recruitment of Treg cells [141].

The miR-200 family inhibits OC angiogenesis by targeting IL-8 and CXCL1, which are secreted by cancer cells and TAECs [233]. Recent studies have demonstrated that OC cell-derived exosomes enhance the angiogenic and migratory capacities of vascular endothelial cells both in vitro and in vivo. Specifically, exosomal miR-92b-3p has been identified as a regulator of tumor-associated angiogenesis through its targeting of SOX4. And overexpression of miR-92b-3p has been reported to enhance anti-angiogenic and anti-tumor capabilities [234]. These findings highlight miRNAs as potential therapeutic targets. Bevacizumab is one of several angiogenesis inhibitors showing satisfactory progression-free survival benefits in Phase III randomized controlled trials for the treatment of OC [235, 236]. Patients with advanced OC may benefit from an assessment of the density of specific TAECs [237].

### Cancer-associated mesothelial cells (CAMs)

The mesothelium, a monolayer of mesothelial cells, provides a protective covering for all organs within the abdominal cavity. This layer is supported by an underlying matrix composed of fibronectin, collagen types I and IV, and laminin [238]. Mesothelial cells can act as a defense barrier against OC metastasis into the intra- and extra-abdominal cavity [239], but have also been shown to establish niches containing tumor cells that facilitate OC metastasis [240], indicative of distinct roles. Factors secreted by cancer and mesothelial cells initially recruit OC cells to mesothelial cells, inducing mesothelial-mesenchymal transition (MMT) in normal cells [241]. CAMs undergo distinct morphological changes compared to their normal counterparts, with disorganization of the polarity of the cytoskeleton [240]. CAMs also exhibit distinct EMT features that that cease to provide a protective function. Instead, they secrete various chemokines that facilitate peritoneal metastasis and contribute to the chemoresistance of OC cells [242, 243].

CAMs secrete high levels of IL-6, which supports OC progression [244]. Mesothelial cells additionally secrete basic fibroblast growth factor, a protein associated with mitosis, angiogenesis and chemotaxis [245]. IL-1 activates CAMs and functions as a source of VEGF in ascitic fluid. In this context, the majority of VEGF is synthesized by resident macrophages and inflammatory cells [246]. Lysophosphatidic acid (LPA) secreted by CAMs has been shown to enhance the adhesion, migration, and invasion of OC cells [247].

Elevated levels of hyaluronic acid (HA) are observed in tumor cells relative to their non-cancerous counterparts, with a particularly pronounced increase in stage III tumors (exceeding 49-fold) and metastatic lesions (exceeding 89-fold) [248]. HA expressed by mesothelial cells promotes tumor cell adhesion via [interactions with] CD44. In research on a mouse model, antibody-induced inhibition of CD44 reduced OC cell adhesion to the peritoneum and spreading capacity [249]. Several studies have confirmed that HA-CD44 interactions promote chemoresistance in different cancer types, such as non-small cell lung cancer carcinoma and multiple myeloma, through multiple signaling pathways [250, 251]. In OC, HA binding to the CD44-Nanog complex activates Nanog target genes Rex1 and Sox2, crucial for maintaining stem cell properties. Activated Nanog interacts with STAT3 to upregulate the multidrug resistance-1 (MDR1) gene, enhancing chemotherapeutic resistance. Additionally, HA facilitates the formation of an ankyrin-MDR1-CD44 complex, promoting drug efflux in OC cells [252]. A separate study consistently found that HA boosts the expression of ATP binding cassette (ABC) transporter proteins in OC cell membranes, leading to increased chemotherapy resistance [253]. OPN secreted by CAMs activates the HA/CD44/PI3K-AKT signaling pathway, promotes ABC transporter protein expression and regulates the BCL-2/BAX ratio, ultimately enhancing resistance to chemotherapy [254]. HA has been recently identified in both stage II/III HGSOC and was shown to enhance the tolerance of cancer cells to cisplatin treatment [255].

OC spherical cells appear more resistant to anticancer drugs relative to monolayers. CAMs promote the formation of spherical shape and motility of OC cells [256, 257]. A mechanistic study found that co-culturing OC and mesothelial cells led to platinum resistance by regulating TGF-β1 and the fibronectin 1/AKT signaling pathways [258]. Further experiments exhibited that overexpression of fibronectin (FN) in CAMs could reduce the sensitivity of OC cells to platinum through activating the Akt signaling pathway [258]. A recent scRNA-seq analysis of 18,403 cells from seven untreated HGSOC patients identified six cellular phenotypes linked to prognosis, revealing that higher CAM levels correlate with poorer outcomes [259]. In addition, VCAM-1 expression on CAMs has been found inversely associated with PFS and OS in OC. Furthermore, platinum resistance is more likely to develop in patients with continuously elevated VCAM-1 expression [260].

### Cancer-associated mesenchymal stem cells (CA-MSCs)

MSCs at tumor sites significantly influence inflammation by secreting various factors and modulating immune function [261]. These cells additionally contribute to inflammation and tumor progression through multiple activities, such as their ability to differentiate into CAFs, suppress immune responses, promote angiogenesis, stimulate EMT, enhance metastasis and inhibit apoptosis [262, 263]. The role of MSCs in cancer remains controversial at present, with evidence of both oncogenic and tumor-suppressive effects [264, 265]. These discrepancies may be due to the MSCs’ origin (cell line, bone marrow, adipose, or tumor) and their exposure to cancer, as local tissue MSCs can be epigenetically reprogrammed by the TME into CA-MSCs [266].

Multiple experimental findings support a direct anticancer activity of MSCs [267–271]. For instance, MSCs can induce tumor necrosis and inhibit cell proliferation in OC through the activity of secreted microvesicles [272]. The anticancer activity of MSCs has been clearly demonstrated *in vitr*o, with significant inhibitory effects on cell growth and migration, along with induction of apoptosis and cell cycle arrest [271, 273, 274]. In vivo experiments have shown that MSCs expressing low levels of CD90 significantly inhibited tumor growth and prolong the survival time of mice. The therapeutic efficacy may be further augmented by the concurrent administration of the immune activator VIC-008, which induces the activation of anti-tumor CD4 + and CD8 + T cells within the TME and concurrently reduces the population of Tregs [275]. In addition, recent studies showed that intraperitoneal injection of conditioned medium from human cervical MSCs inhibits tumor growth and extends survival in mice [276].

Conversely, a substantial body of research has corroborated that adipose-derived mesenchymal stem cells (ADSCs) located within omental adipose tissue facilitate the proliferation of OC cells and induce a transition towards a more invasive and metastatic phenotype [277–281]. Co-culturing OC cells with MSCs activates genes linked to proliferation, migration, invasion, and drug resistance [282]. Co-culturing OC cells with ADSCs has been shown to enhance OC cell proliferation and invasion by increasing PAX8 and TMSB4X levels, which are crucial for cancer cell growth [283, 284]. Another study on the interactions of omental ADSCs with OC cells demonstrated that upon co-culture, ADSCs promoted significant invasion and proliferation of OC cells by stimulating the secretion of MMPs [281]. Moreover, CD44 on ADSCs interacts with MMPs, influencing ECM remodeling and aiding cancer cell infiltration. Blocking MMP2 and MMP9 may reduce ADSCs’ proliferative and invasive impact on OC cells [285].

CA-MSCs associated with OC exhibit elevated proteogenic activity, which imparts resistance to chemotherapy when co-implanted with OC cell lines [286]. Ovarian CA-MSCs are highly proteogenic and cause chemoresistance when co-implanted with OC cell lines and primary ovarian tumor cells [264, 286]. ADSCs derived from human omentum enhance the resistance of OC cells to paclitaxel or carboplatin [278]. Importantly, ADSCs also promote chemoresistance partly via nitric oxide pathway modulation [287]. ADSCs enhance autophagy in OC cells, which is reported to contribute to chemoresistance [288, 289]. Furthermore, ADSCs inhibit caspase-3 cleavage and reduce cisplatin-induced apoptosis and platinum levels, thereby promoting chemoresistance in OC cells [290]. Conditioned medium of ADSCs from metastatic omentum of OC patients is reported to stimulate cytokine and promote greater chemoresistance to cisplatin and paclitaxel compared to non-metastatic omentum ADSCs [291].

MSC-based cancer therapy is currently of significant research interest due to the remarkable tumor homing properties of MSCs [292, 293]. Bone marrow-derived MSCs are known to migrate to primary cancers or metastases following systemic infusion [294]. The exosomes and membranes derived from MSCs possess the capability to deliver chemotherapy agents, therapeutic genes, and oncolytic viruses with high specificity to target and eradicate cancer cells [295–298]. The debate on whether MSCs are anticancer agents or targets for cancer therapy is ongoing. Only two clinical trials (NCT02530047(Registration Date: 2015-08-19) and NCT02068794) have explored MSCs for OC treatment, but no results have been published.

### Exosomes

Exosomes are widely present in the TME and considered essential regulators of the microenvironment and tumor progression [299]. By releasing bioactive molecules, exosomes have a substantial impact on several pathways, including tumor angiogenesis, cellular signaling and communication, immune regulation, tumor metastasis, and chemoresistance [300], and may therefore serving as potential biomarkers of OC cell growth, spread, and immune evasion [301].

CAF-derived exosomes co-cultured with OC cells induce malignant behaviors, including increased migration and invasion potential and promotion of the EMT through activation of the small mother against decapentaplegic (SMAD) signaling pathway [302]. OC exosomes transform fibroblasts into CAFs, boosting TGF-β1production and activating SMAD signaling mutations. In hypoxic conditions, tumor cells release more exosomes with increased angiogenic and metastatic capabilities, stimulating alterations in the TME and tumor progression [303]. And TGF-β1 in CAF-derived exosomes promotes a more invasive phenotype of OC cells, supporting the potential utility of targeting CAF-derived exosomes as a therapeutic approach for OC.

A previous study found that exosomes from MA in patients with OC contained two cargo proteins, epithelial cell adhesion molecule (EpCAM) and CD24. Whereas increased concentrations of EpCAM were associated with the stage of OC, CD24 was a reliable indicator of poor outcomes in OC. and other cancers [304]. Exosomes secreted by OC cells induce T-cell arrest, thereby facilitating the immune evasion of cancer cells [305]. In addition, exosomes are able to evade immune surveillance through inhibiting NK cell function [306], suppressing differentiation of DCs [307] and promoting differentiation of myeloid suppressor cells [308]. Additionally, OC exosomes trigger apoptosis in DCs, hematopoietic stem cells, and peripheral blood lymphocytes, thereby suppressing anti-tumor immune responses [309].

Exosomes may further contribute to treatment resistance in OC. Increased expression of Annexin A3 in exosomes released from cisplatin-resistant OC cells is linked to platinum resistance [310]. In addition, cancer-derived exosomes can deliver CRISPR/Cas9 to OC cells, suppress PARP-1, trigger apoptosis, and increase cisplatin sensitivity [311]. Exosomes with plasma gelsolin (pGSN) (Ex-pGSN) have been shown to influence OC chemosensitivity. These experiments demonstrated that Ex-pGSN enhances HIF1α-mediated pGSN expression in chemoresistant OC cells through autocrine signaling, and also imparts cisplatin resistance to chemosensitive OC cells [312]. A subsequent investigation conducted by the same research group revealed that elevated levels of exosomal pGSN produced by chemoresistant OC cells induced CD8 + T cell apoptosis and reduced γ-interferon secretion, supporting the theory that exosomal pGSN promotes chemoresistance through immune surveillance [313].

Exosomal miRNAs are unequivocally implicated in the processes of tumorigenesis, metastasis, and the development of drug resistance [314–316]. Exosomes isolated from CAFs and CAAs express significantly higher levels of miR-21, which suppresses APAF1 protein in neighboring tumor cells and increases chemoresistance to paclitaxel [213]. In addition, CAF-derived exosomes promote cisplatin resistance in OC by inhibiting the delivery of miR-98-5p by CDKN1A, a key regulator of cell cycle arrest and apoptosis [214]. miR-1246 expressed in OC exosomes is reported to trigger resistance to paclitaxel through the Cav1/multidrug resistance protein 1 (p-gp)/M2 phenotype macrophage axis [317]. In addition, exosomal miR-21-3p inhibits the expression of the protein-coding gene neuron navigator 3 in A2780 cells and cisplatin-resistant variant CP70 cells, thereby enhancing resistance to cisplatin [318]. Exosomal miR-433 enhances paclitaxel resistance by causing cellular senescence and suppressing the proliferation of nearby cells [319]. Recent reports show that exosomal miR-429 boosts proliferation and drug resistance in A2780 cells and mouse tumors by targeting the calcium-sensing receptor/STAT3 pathway [320]. Furthermore, Exosomal miR-223 from hypoxic macrophages boosts OC cell drug resistance via the PTEN-PI3K/AKT pathway, both in vitro and in vivo. It could also be a biomarker for chemotherapy response and a target to overcome chemoresistance in advanced OC patients [321].

Exosomes are also considered valuable carriers for drug delivery. Exosomes derived from expanded natural killer cells (eNK-EXO) exhibit characteristic protein markers typical for preferential uptake by SKOV3 cells, inducing cytotoxicity in OC cells. Moreover, eNK-EXO can be utilized to deliver cisplatin, enhancing its cytotoxic effects on drug-resistant OC cells and reversing the immunosuppression of NK cells. These findings underscore the significant potential of eNK-EXO for clinical application in the management of OC [322]. Exosome-mediated TME regulation not only relies on intercellular communication, but also affects tumor progression through ECM remodeling, which will be discussed in detail in the next section.

### ECM

The ECM, integral to OC development and progression, comprises collagen fibers for strength, proteoglycans for cell shelter, and adhesive glycoproteins (like laminin and fibronectin) that connect collagen and proteoglycans to cell receptors (e.g., integrins, hyaluronic acid receptors) [323].The ECM component supports tumor development through providing proliferative signals, facilitating evasion of tumor growth inhibitors and apoptosis, enhancing replicative immortalization, inducing neovascularization and promoting tumor cell invasion and metastasis [324]. In OC, high expression of ECM is significantly linked to poor immune status and low patient survival [325].

Under hypoxic conditions, mesothelial cells have been observed to secrete lysyl oxidase, an ECM remodeling enzyme that facilitates the crosslinking of collagen fibers, thereby forming fibrillar collagen. This process of ECM remodeling plays a significant role in promoting tumor invasion in HGSOC [326]. ADAM23, the member of a disintegrin and metalloproteinase (ADAM) family, is a significant focus of attention, owing to its expression in many tumor types. A significant correlation exists between tumor stage, lymph node metastasis, and reduced PFS and OS in OC patients lacking ADAM23 expression. Furthermore, ADAM23 has been identified as an independent predictor of survival in OC patients [327]. Notably, β1 integrins interact with nearly all common ECM components [328], are overexpressed in OC and linked to poor outcomes [329, 330]. Urokinase-type plasminogen activator (uPA) and its inhibitors, plasminogen activator inhibitor type-1 and type2 (PAI-1 and PAI-2), are crucial in tumor invasion and metastasis. Over 75% of OCs show high levels of uPA and PAI-1, which are linked to chemotherapy resistance, advanced tumor stage, poor differentiation, residual disease, and increased invasiveness [331, 332].

At the same time, the ECM can induce chemoresistance in OC cells by initiating a metabolic shift that relies on fatty acids as an energy source. For instance, the serine protease kallikrein-related peptidase (KLK) family is notably upregulated in OC [333]. Specific KLK7 isoforms in HGSOC promote chemoresistance through multicellular aggregation [330]. A recent study found HA and fibronectin in stage II/III HGSOC patients and two cancer cell lines (OVCAR-3 and SKOV-3). The study demonstrated that HA enhances the resistance of cancer cells to cisplatin treatment, while fibronectin facilitates cancer cell proliferation and invasion by inducing ERK and p38 signaling pathways [255]. A supplementary investigation demonstrated that COL11A1 activation of Akt/c/EBP signaling pathways resulted in the stabilization of pyruvate dehydrogenase kinase isoform 1 (PDK1) in OC cells, thereby imparting resistance to cisplatin and paclitaxel [334]. Interestingly, inhibition of these anti-apoptotic proteins re-sensitized OC cells to cisplatin, highlighting potential therapeutic utility as targets for recurrent OC with high COL11A1 expression [335].

The preceding sections have outlined how TME components such as immune cells, adipocytes, and CAFs drive OC progression. Next section integrates these elements to illustrate their synergistic role in peritoneal metastasis, a hallmark of OC.

## TME with OC peritoneal metastasis

**Fig. 3:**
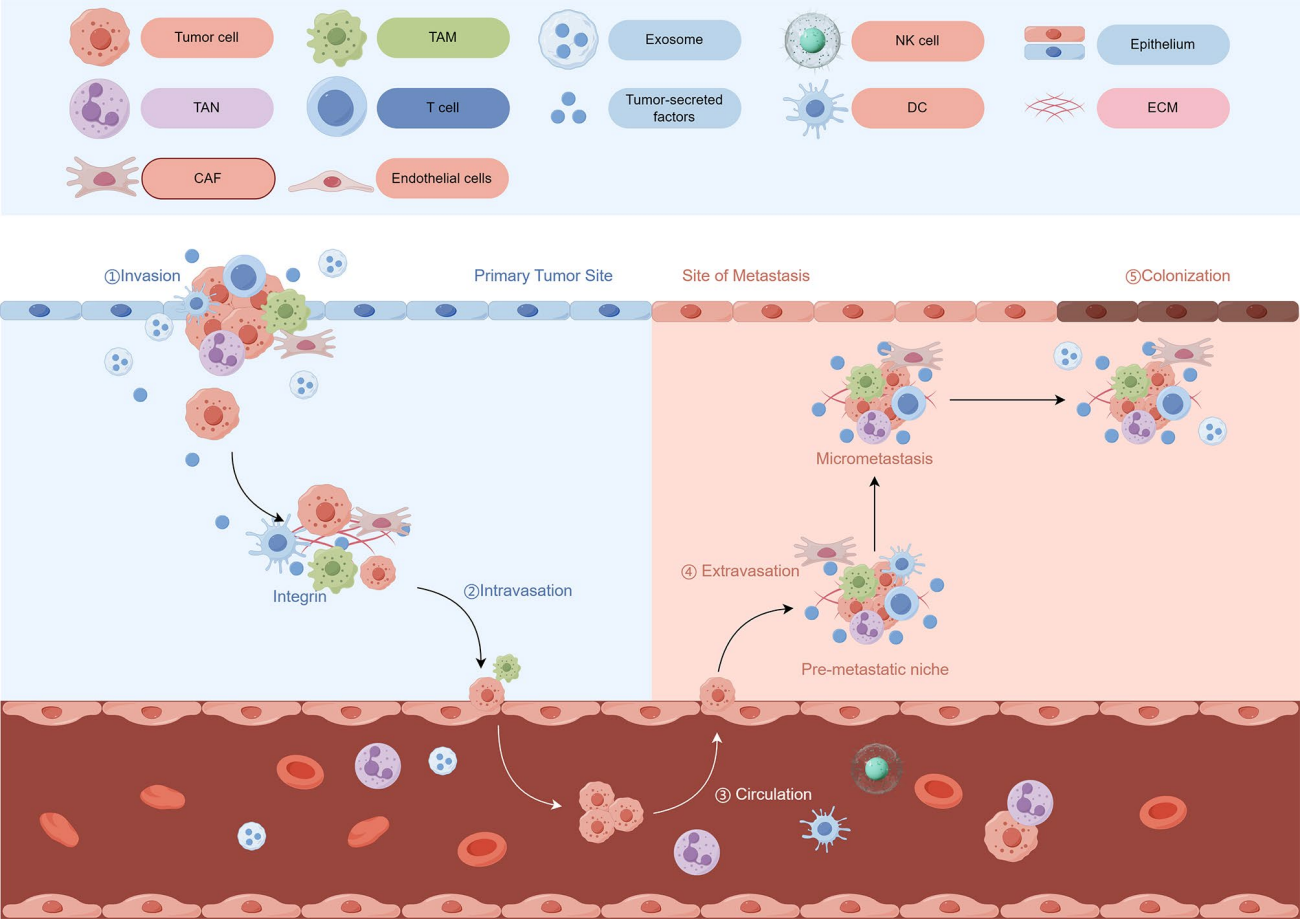
Primary tumor progression and metastasis and complex interactions within the TME. TME evolves throughout the various stages of cancer progression. The TME includes a variety of immune cells, cancer-associated fibroblasts, endothelial cells and extracellular matrix. These components may vary by tissue type and co-evolve as the tumor progresses. The cells and factors of the TME also play an important role in preparing the pre-metastatic ecological niche as well as facilitating extravasation. During the metastatic phase, TME helps to control metastatic cell dormancy, emergence from dormancy, and subsequent metastatic growth. TME, tumor microenvironment

Due to the complexity of the peritoneal environment in OC, the omentum becomes the optimal matrix for promoting and maintaining metastasis (Fig. 3). Currently, two hypotheses have been proposed for the peritoneal metastasis model of OC. The first hypothesis, related to the “seed and soil” hypothesis [16], is that peritoneal metastasis of OC originates from circulating tumor cells in the peritoneal cavity, which preferentially metastasize to the peritoneum via the transurethral, hematogenous or lymphatic pathways. The second hypothesis is known as the metaplasia hypothesis, which states that the metastatic peritoneal site of OC is a synchronous malignant transformation of the peritoneum or omentum, as there is a similar lineage between ovarian epithelium and omentum [4]. Although further research is needed to understand how peritoneal metastases develop, the “seed and soil” hypothesis has been widely accepted historically [336], suggesting multiple interactions between metastatic cells and certain homeostatic mechanisms specific to the microenvironment of certain organs. The affinity of tumor cells (“seeds”) for a specific organ environment (“soil”) is a key factor in determining whether metastases can form. The peritoneal tissue becomes ideal soil for the implantation and metastasis of OC cells. Cancer cells passively shed from the primary tumor into the peritoneal cavity, where they are carried by peritoneal fluid to the peritoneal surface, leading to multifocal metastases [337].

The exact mechanisms underlying the dissemination of OC with pronounced tropism, in relation to the intricate interactions between tumor and stromal cells within the TME, remain inadequately understood. In this section, the functions of components of the TME in peritoneal metastasis are summarized and how the TME in the peritoneal cavity supports OC peritoneal metastasis.

### TIME and OC metastasis

TAMs in OC exhibit dual localization in the primary tumor and metastatic omentum, with M1/M2 imbalance contributing to peritoneal metastasis progression [338, 339]. Importantly, the large spheroid population in OC patients is heterogeneous and consists of TAM-OC cells [29]. M2-like TAMs are predominantly situated at the core of the spheroids and are implicated in the mechanisms that facilitate tumor cell proliferation and migration during OC metastasis [29, 340, 341].

In general, TAMs promote the metastasis of OC by producing a variety of mediators. A study found that TNF-α released by M1-like TAMs increase the metastatic potential of OC cells by activating the NF-κB signaling pathway [342]. In addition, Hagemann and colleagues demonstrated that macrophages induce invasiveness of epithelial cancer cells via nuclear factor-κB and c-Jun NH2-terminal kinase signaling [25]. Furthermore, studies have shown that M2-like omental macrophages secrete several critical proangiogenic factors and ECM remodeling proteins, such as TGF-β, VEGF-C, and MMP9, which facilitate sphere implantation [11, 343]. In addition, M2-like TAMs can promote peritoneal metastasis by activating CCR5/PI3K signaling to promote the adhesion of tumor cells to mesothelial cells [344]. M2-like TAMs have strong paracrine activity and significantly contribute to the establishment of the immunosuppressive TME, promoting tumor growth, angiogenesis, invasion and further metastatic dissemination [345].

Studies utilizing murine models have revealed that TAMs constitute a significant cellular component of the intra-abdominal milieu. Furthermore, TAMs are crucial for the trans-intestinal dissemination of ovarian tumor cells, thereby facilitating their survival and invasiveness [29, 346]. TAMs promote pre-metastatic niche formation and eosinophilization of OC cells through the release of associated soluble factors, which contribute to the growth and peritoneal metastasis of tumor cells [347, 348]. Moreover, TAMs enhance metastasis by impairing T cell function [349]. M2-like TAMs polarization can also inhibit metastatic colonization of OC by stabilizing WAP four-disulfide core domain 1 and IL-17D inhibition by sorbitol and SH3 domain containing 2 [350].

Tumor-associated neutrophils (TANs) additionally play a role in OC metastasis. For instance, TANs within breast plaques contribute to the formation of pre-metastatic omental niches, promoting the implantation and colonization of OC cells in the omentum [351]. Furthermore, patients diagnosed with advanced OC, exhibit elevated baseline neutrophil-to-lymphocyte ratio (NLR), which are significantly correlated with the presence of distant metastases [61, 352]. Another recent study reported the involvement of Myeloid-derived suppressor cells (MDSCs) in EMT or formation of “pre-metastatic niches” [353]. Tumor-resident MDSCs were shown to increase the metastatic potential of OC by triggering expression of miRNA101 in OC cells, which, in turn, targeted the 3’-UTR region of the co-repressor gene C-terminal binding protein-2 and disrupted its binding to promoters of NANOG, OCT4/3 and SOX2, key genes involved in maintaining the pluripotency of primary OC cells [354]. In addition, a study found that MDSCs inhibited T cell activation and enhanced gene expression, OC stem cell sphere formation, and metastasis. MDSCs in the TIME secrete PGE2 to activate the intracellular miRNA101 or CSF2/STAT3 pathway, causing OC cells to acquire stem cell properties and increase PD-L1 expression, thereby supporting OC cell immune escape [354].

T lymphocytes are a critical component of the adaptive immune system crucial for the clearance of tumor cells by the host immune system. The CD4 + T cell population is increased in ascites of OC patients compared to primary sites and peritoneal metastases [122, 156]. Additionally, Tregs suppress anti-tumor immunity, and their buildup in OC ascites correlates with advanced disease stages [138]. Tregs release TGF-β, leading to a tumor-promoting microenvironment and formation of tumor cell EMT [355]. Multiple cytokines and chemokines are additionally associated with peritoneal metastasis of OC. These molecules accumulate to create a pro-inflammatory and immunosuppressive TME that promotes peritoneal colonization and neovascularization of developing tumor implants [356, 357]. For example, LPA, a growth factor overexpressed in OC ascites, promotes proliferation and migration of OC cells [358]. A recent study found that increased levels of the chemokine CXCL8 in the OC TME promote tumor growth, spread, and peritoneal metastasis. CXCL8 also interacts with peritoneal metastases to further enhance progression [359].

### CAAs and OC metastasis

Adipocytes are integral to the process of omental metastasis [163]. Adipocytes in peritoneal metastases supply ample nutrients for tumor cell growth, facilitate their initial homing to the omentum via secretion of adipokines, and subsequently provide fatty acids to promote rapid tumor growth [163]. Additionally, adipocytes can metastatically colonize the omentum when co-cultured with non-HGSOC tumor cell lines, as supported by in vivo xenograft models [163]. Furthermore, adipocytes facilitate the uptake of fatty acids and enhance energy metabolism in OC cells by increasing CD36 expression on their surface, thereby contributing to peritumoral metastasis [360]. Cysteine-rich acidic secretory proteins conversely alleviate peritoneal metastasis of OC by inhibiting adipocyte differentiation and interactions between adipocytes and tumor cells [170].

The adipose microenvironment can facilitate the migration process by producing a variety of adipokines, growth factors and hormones. IL-6, IL-8, and monocyte chemotactic protein-1 released by the omentum promote the dissemination of OC cells [163]. Moreover, omental-derived IL-8 activates the p38 MAPK/STAT3 axis via CXCR1 in OC cells, which promotes OC metastasis. Additionally, conditioned media from CD45-/CD31- adipose stromal cells from subcutaneous or visceral fat activates the JAK2/STAT3 pathway via IL-6, enhancing OC cell migration [361]. A study by Tong and co-workers [362] revealed that exogenously added IL-6 activated the JAK2/STAT3 pathway and promoted migration of OC cell lines. In vitro IL-8 knockdown inhibited OC cell migration, and in vivo, IL-8 and IL-6 neutralizing antibodies prevented OC cells from homing to the adipose microenvironment in a xenograft model [163]. IL-33 has additionally been reported to activate ERK signaling and promote OC cell migration and invasion [363]. Finally, MCP-1 from adipocytes may trigger OC cell migration and omental metastasis by binding to CCR-2, activating the PI3K/AKT/mTOR pathway and downstream HIF-1α and VEGF-A [364, 365].

Although OC exhibits a unique metastatic pattern compared to other solid tumors, the EMT process remains a key step in its genesis [366, 367]. Adipocytes induce alterations in adhesion and tight junctions as well as the cytoskeleton, thereby triggering the EMT. In addition to preserving the epithelial phenotype and facilitating cell-cell interactions, E-cadherin is integral to the function of adherens junctions [368]. E-cadherin deficiency is predictive of lower OS in patients with OC [369, 370]. In addition, adipose tissue produces a number of soluble growth factors, such as HGF, IGF-1, and FGF, which are implicated in the formation of EMT [371–373]. HGF produced by adipose-rich tissues is associated with the absence of E-cadherin and promotes the migration of several OC cell lines in vitro [374]. Consistent with this finding, a neutralizing antibody against HGF inhibited migration of the SKOV3 cell line [375]. Another in vitro study using OC cell lines showed that IGF-1 inhibited E-cadherin expression through modulation of the PI3K/Akt/mTOR signaling pathway [376]. Furthermore, FGF promotes the downregulation of E-cadherin through the activation of the PI3K/Akt/mTOR and MAPK/ERK signaling pathways in human OC cells [377].

Treating OC cells with LEP activates EKR and JNK pathways, inducing MMP-7, -2, and − 9, which enhance migration [378]. This finding is in keeping with a separate report that LEP induces MMP-7 and promotes OC invasiveness through activation of ERK and JNK pathways [379]. Similarly, LEP treatment has been shown to promote the migration and wound healing capacity of OC cell lines [172]. Additionally, TNF-α secreted by adipocytes induces CD44 expression in OC cells through activation of JNK pathway [380]. The pivotal role of CD44 in fostering a pro-tumorigenic microenvironment, as well as in promoting angiogenesis, immunosuppression, and metabolic reprogramming in OC, has been underscored [381]. Compared with primary OC, FABP4 levels are found elevated in peritoneal metastases. FABP4 deficiency significantly inhibits the growth of metastatic tumors in mice, indicating that FABP4 plays a key role in OC metastasis. FABP4 synthesis in adipocytes appears to be a key step in the transfer of fatty acids to cancer cells and contributes to angiogenesis and tumor proliferation [382].

Thus, modulating the adipose microenvironment has the potential to influence metastatic progression at every stage, leading to epigenetic changes that effectively enhance OC migration and invasion. Understanding the role of adipocytes in OC peritoneal metastasis is crucial for enhancing diagnosis and treatment.

### CAFs and OC metastasis

Activated CAFs appear shortly before the invasive tumor stage of most cancer types and promote proliferation and metastasis by remodeling the ECM scaffolding as well as stimulating the production of paracrine growth factors and chemokines [187]. CAFs are involved in multiple signaling pathways in tumor promotion, which exerts a pivotal influence in inducing angiogenesis at the tumor site as well as increasing tumor cell proliferation and migration in different cancer systems [383, 384]. A study identified VCAN as a key upregulated gene in CAFs that promotes the motility and invasion of OC cells by activating the nuclear factor-κB signaling pathway and upregulating CD44, MMP-9, and hyaluronan-mediated motility receptor expression in cancer cells. They found that VCAN expression in CAFs is regulated by the activation of TGF-β signaling in CAFs induced by TGF-β ligand secretion from OC cells. The cross-talk between cancer cells and CAFs via VCAN plays a key role in the progression of OC under TGF-β stimulation [194].

In addition, CAFs can accelerate OC progression through either direct or indirect effects. These cells secrete a variety of cytokines that enhance peritoneal metastasis. For example, CAF-induced elevation of VEGF-A and IL-6 is reported to promote peritoneal metastasis through activating the EMT mechanism [385]. Likewise, a study confirmed that CAF-derived CXCL12 induces EMT via the CXCR4/Wnt/β-catenin pathway in OC cells [218]. At the same time, CAF-derived microfibrillar-associated protein 5 (MFAP5) binds to the α_V_β_3_ integrin receptor on the surface of OC cells, activating the Ca^2+^-dependent FAK/cAMP response element binding protein/type 1 myosin C signaling pathway [386]. Activation of this signaling pathway stimulates the reorganization of the F-actin cytoskeleton and enhances the production of cell traction forces, thereby increasing the migration potential of OC cells. In addition, CAFs secrete EGF and maintain the expression of integrin α5 (ITGA5) on HGSOC ascites tumor cells (ATCs). ATCs with elevated ITGA5 form diverse spheroids with CAFs, promoting early peritoneal spread of HGSOC and faster ascites development [387].

HO-8910pm, a metastatic OC cell line, promotes tumor proliferation, adhesion and migration through upregulation of fibroblast activating protein-1α [388]. Additionally, human and mouse omental CAFs are stimulated by discoidin domain receptor 2 (DDR2), which enhances collagen synthesis through the activation of arginase. CAFs with high levels of DDR2 or arginase are associated with enhanced tumor colonization of the omentum [389]. Another study suggests that the IL-33/ST2 axis in OC integrates IL-33-expressing CAFs with M2-like CAMs to exacerbate invasion and metastasis via EMT progression [390]. Furthermore, CAFs overexpressing Glis Family Zinc Finger 1 have been shown to support migration and metastasis of OC cells [391], highlighting the potential of GLIS1 in CAFs as a therapeutic target for limiting OC metastasis. Other factors that additionally contribute to metastasis, such as urokinase-type activator of fibrinogen and the pro-inflammatory factors CXCL-1 and COX-2, are secreted by CAFs [392]. In summary, CAFs stimulate self-migration and angiogenesis, facilitating the survival, proliferation and invasion of tumor cells.

### Endothelial cells and OC metastasis

Angiogenesis plays a crucial role in the peritoneal spread of OC [393]. Once a tumor metastasizes, new blood vessels must form to supply nutrients for tumor cell survival and dissemination. Cells in the TME, such as macrophages, tumor cells, and mesothelial cells, attract peritoneal endothelial cells to the metastatic site. They promote implantation and progression by secreting chemokines, TGF-β, and IL-6, which help form tube-like structures [394–396]. Additionally, VEGF promotes angiogenesis and vascular permeability in peritoneal ECs, leading to ascites and a metastasis-friendly environment [397].

There is a higher expression of VEGF-A, VEGF-D, and VEGFR1 in ovarian metastases compared with primary ovarian epithelial tumors [398]. Angiogenesis induced by VEGF may enhance the growth of large metastatic nodules at the site of metastatic lesions. In addition, the finding that VEGF inhibits T cell activation and proliferation supports a mechanism of VEGF-mediated enhancement of metastasis in OC through effects on immune cell function [399]. VEGF expression in omental metastases correlates with the extent of involvement and independently predicts prognosis. High VEGF, TGF-β, and IL-6 levels in ascites are linked to shorter PFS [400, 401].

Several studies have examined the role of VEGF and MMPs in OC peritoneal spread. One earlier study highlighted a link between VEGF levels and MMP-2 expression and activation, suggesting this relationship is tied to peritoneal progression [402]. Another investigation by Belotti et al. [403] revealed that MMPs, particularly MMP-9, facilitate the release of biologically active VEGF, thereby contributing to the development of ascites. Furthermore, VEGF promotes organ-specific MMP-9 expression, and its inhibition lowers MMP-9 levels, preventing ascites and reducing intraperitoneal tumor load [404].

Deletion of Smad4 (a key factor involved in the response to TGF-β-related ligands) in endothelial cells disrupts the integrity of the endothelial cell barrier and increases vascular permeability, thereby promoting OC metastasis [405]. Apoptosis signal-regulated kinase 1 (ASK1) can mediate degradation of the endothelial junction protein VE-cadherin via the lysosomal pathway to promote macrophage migration. Inhibition of ASK1 expression has been shown to attenuate vascular permeability, TAM infiltration and transmucosal metastasis of OC cells in a mouse model [406]. Furthermore, the expression of Notch1 receptors (N1ICD) in tumor endothelial cells facilitates peritoneal metastasis and correlates with reduced survival in a murine model of OC. Activated N1ICD induces endothelial cell senescence, upregulates VCAM-1 expression, promotes neutrophil recruitment, and enhances tumor invasion [407].

Other known angiogenic factors include fibroblast growth factor (FGF) and its transmembrane tyrosine kinase receptor (FGFR) [408]. One study showed that overexpression of FGFR4 (one of the key receptors for FGF1) in OC cells was associated with poor patient survival [409]. In addition, silencing FGFR4 in OC cells significantly inhibited FGF1-activated mitogen-activated protein kinase, nuclear factor-κB and WNT signaling pathways. Silencing FGFR4 by FGFR4-specific small interfering RNA and blocking FGFR4 activation by FGFR4-capturing protein effectively inhibited the in vivo growth of OC [409].

### CAMs and OC metastasis

Mesothelial cells are the initial barrier for metastatic OC cells. These cells enhance adhesion, growth, and invasion of HGSOC tumor cells, indicating a role in ovarian peritoneal metastasis [410]. Once MMT occurs, mesothelial cells induce tumor cell invasion through enhancing adhesion to the peritoneum [238] and accumulation of CAFs [411]. In addition, CAMs secrete fibronectin and provide access to the subepithelial ECM, facilitating initial metastatic colonization of OC cells [410].

During peritoneal metastasis of OC, CAMs regulate cytokine expression to aid tumor cell adhesion and invasion. A recent study found that OC patients have significantly lower levels of intelectin-1 (ITLN1) in CAMs and serum compared to healthy women. Additionally, fusing ITLN1 with lactotransferrin (LTF) inhibited LTF’s binding to the low-density lipoprotein receptor-related protein 1 (LRP1) on OC cells. ITLN1 attached to LRP1 and induced transcriptional activation of MMP1 expression, thereby promoting cancer cell invasion and metastasis [412]. Furthermore, in OC, the hypoxic microenvironment is reported to promote the deposition of extracellular collagen fibers by CAMs and cancer cells in a HIF-1- and HIF-2-dependent manner, ultimately leading to early metastasis and tumor invasion [326].

Various cytokines secreted by CAMs additionally play a role in OC metastasis. IL-8 produced by CAMs induces overexpression of PDK1 in OC cells through CXCR1 interactions. TME-regulated PDK1 promotes OC metastasis by regulating tumor-mesothelial adhesion, invasion, and angiogenesis through α5β1 integrin and JNK/IL-8 signaling [413]. Furthermore, IL-8 binding to CXCR1/CXCR2 on endothelial cells has been proven to promote tumor neovascularization [414]. CAMs generate LPA via calcium-independent phospholipase A2 (iPLA2) and cell membrane phospholipase A2 (cPLA2) activities, which stimulate kinase and Akt signaling pathways in OC cells. This promotes tumor cell adhesion to collagen I, leading to metastasis [247]. In addition, it was demonstrated that peritoneal mesothelial cells in the TME of OC patients secrete the non-canonical Wnt ligand Wnt5a. Wnt5a promotes the adhesion of OC cells to peritoneal mesothelial cells and promotes their migration and invasion, leading to the colonization of peritoneal transplant tumors. They found that tumors formed in Wnt5a knockout mice had high levels of cytotoxic T cells, high levels of M1 macrophages, and low levels of M2-like TAMs, indicating that host Wnt5a promotes an immunosuppressive microenvironment. Src family kinase Fgr was identified as a downstream effector of Wnt5a. These results highlight the role of host-expressed Wnt5a in OC metastasis and suggest that Fgr is a novel target for inhibiting OC metastasis progression [415].

Complex interactions between CAMs and cancer cells contribute to metastasis, such as TGF-β from OC cells, leading to metastasis of mesothelial cells to CAMs [416]. CAMs boost VEGF secretion in a TGF-β-dependent way, enhancing the migration and duct formation of subperitoneal endothelial cells, thus promoting tumor neovascularization [417]. Furthermore, TGF-β triggers the RAC1/SMAD3 pathway by attaching to TGF-bRII, leading to an increase in fibronectin levels in CAMs. This fibronectin, present in the ECM, connects with α5 and β1 integrins found on OC cells, consequently promoting metastasis [410]. Moreover, OC cells excessively produce PAI-1 and DLX4, which trigger IL-8/CXCL5 and IL-1b/CD44 expression via NF-kB signaling in CAMs, intensifying tumor-cell interactions and metastasis [418, 419].

Senescent mesothelial cells promote adhesion of tumor cells to the peritoneum and aid in the establishment of peritoneal metastases in OC [420]. FN is upregulated and connectivity proteins (such as E-cadherin) downregulated in these cells, leading to disruption of peritoneal mesothelial cell integrity and higher invasiveness of OC [421]. Aging mesothelial cells also release factors that promote angiogenesis, like CXCL1, CXCL8, and VEGF, thereby encouraging neovascularization in subperitoneal tumors [395].

### MSCs and OC metastasis

MSCs are critical for the metastatic microenvironment of the OC omentum. ADSCs and CAFs within the OC microenvironment regulate cancer cell behavior, including adhesion, survival, proliferation, and metastasis. Their presence and transformation into CAFs due to TGF-β1 are crucial for encouraging OC growth, survival, EMT, and the development of a cancer stem cell-like phenotype [422]. Similarly, ADSCs located in the TME could stimulate OC growth and metastasis via activation of EMT and TGF-β signaling [279]. Specifically, omental ADSCs promote tumor angiogenesis and OC cell survival by secreting VEGF and SDF1-α [345]. Additionally, ADSCs are known to release elements such as IL-1 receptor antagonists, IL-6, IL-10, CCL5, VEGF and MMP-2, which have been associated with metastatic aggression in OC [345]. A metastasis-promoting role of ADSCs through production of MMP2 and MMP-9 proteins has been demonstrated in a mouse xenograft model [281]. A recent study revealed a unique epigenetic landscape of CA-MSCs compared to their normal MSC counterparts. Interestingly, the direct interaction between CA-MSCs and tumor cells resulted in the advancement of metastasis in OC. This was accomplished via a co-metastatic process, whereby the CA-MSCs and tumor cells collaborated in their movement to successfully colonize the metastatic site [423]. Another study demonstrated the important role of CA-MSC in enhancing OC heterogeneity through horizontal mitochondrial transfer. After receiving mitochondria donated by CA-MSC, tumor cells undergo transcriptional changes that amplify the effects of mitochondrial transfer by secreting angiopoietin-like 3 and activating the MAPK/ERK signaling pathway to promote OC proliferation [424].

### Exosomes and OC metastasis

Earlier research suggests that exosomes in the TME influence OC cell invasion and metastasis. These extracellular vesicles promote peritoneal spread of OC by mediating cell-to-cell communication. Exosomes produced by ascites facilitating the forming of metastatic anterior niches in the peritoneal cavity and EMT of tumor cells [425], and play important roles in the progression of OC. Furthermore, exosomes interact with other cells and act as carriers of proteins and RNA (mRNA or miRNA) for intercellular transfer. Exosomal miRNAs exert an instructive role in pre-translocation ecology [426].

MAs from OC patients contain tumor-associated exosomes with potentially crucial roles in cell signaling and ECM protein degradation. Protein hydrolases have been isolated from these exosomes, suggesting a role in promoting migration and invasion of OC cells during the metastatic process [427]. Exosomes in the ascites of OC patients have been shown to transport miR-6780b-5p into OC, which is associated with tumor metastasis. This promoting function is based on the fact that miR-6780b-5p overexpression promotes EMT in OC cells [425]. In the omental TME, exosomes secreted by stromal cells containing miR-21 could alter the invasive phenotype of metastatic OC cells, signifying a novel directional strategy for inhibiting metastasis [213]. In addition, miR-21 targets the tumor suppressor programmed cell death gene 4 (PDCD4) and plays a contributory role in malignant transformation. Sustained overexpression of miR-21 and deletion of PDCD4 may lead to tumor spread [428, 429]. Under a hypoxic microenvironment, high expression of miR-940 in exosomes of OC cells is reported to induce macrophage differentiation to an M2 phenotype, promoting tumor proliferation and metastasis [430]. Similarly, Exosomal miR-99a-5p is elevated in sera of OC patients and promotes cancer cell invasion by increasing fibronectin and vitronectin expression in neighboring peritoneal mesothelial cells [431]. In addition, a study showed that tumor-derived miR-205 can be transported from OC cells to macrophages via exosomes, and promote cancer cell metastasis by inducing M2-like macrophage polarization and activating the PI3K/AKT/mTOR signaling pathway [432].

Another study confirmed that exosomes actively facilitate peritoneal dissemination by remodeling the TME. Following co-culture of OC-derived exosomes with peritoneal mesothelial cells, fluorescent labeling and tracking revealed that cell surface glycoprotein CD44 was transferred and mesenchymal morphology induced in these cells. Moreover, the cells acquired an invasive phenotype [433]. Exosomes from OC patient ascites contain activated matrix urokinase, MMP-9, and MMP-2, fibrinogen activator, promoting protease activation, ECM degradation, and cell migration and invasion [434]. In addition, macrophage-derived exosomes stimulated by TNF-related weak inducers of apoptosis (e.g. TWEAK) can be internalized by tumor cells, leading to inhibition of OC metastasis. TWEAK stimulation reportedly boosts miR-7 expression in macrophage-released exosomes, subsequently inhibiting the EGFR/AKT/ERK1/2 signaling pathway and decreasing OC metastasis [435].

### ECM and OC metastasis

Tumors use ECM remodeling to create a microenvironment that facilitates tumorigenesis and metastasis. In OC, significant omental metastases involve extensive ECM alteration. Both cancer and mesenchymal stromal cells induce a fibrous tissue growth response, turning the fatty omentum into hard fibrotic tissue. This aligns with the cancer cell-driven breakdown of fat cells, promoting tumor growth [325, 436]. A proteomic study of OC interactions with peritoneal cells highlighted a key link between the annexin A2 signaling pathway and activation of the plasminogen-plasmin system. They observed that OC interactions with peritoneal cells degrade multiple ECM proteins, including fibrinogen, POSTN, annexin A2 and PAI-1. These proteins promote OC cell adhesion to the peritoneum and metastatic colonization via the plasminogen-plasmin pathway, and their mRNA levels can predict prognosis, with elevated levels in the most metastatic and poorest prognosis OC subtype [437].

In epithelial peritoneum and omentum, collagen and fibronectin, which are plentiful ECM proteins, attach to integrin receptors found on OC cells. The precise roles of these proteins in early omental and peritoneal metastasis have been extensively investigated [438, 439]. Overexpression of fibronectin, which contributes to OC cell adhesion, invasion, proliferation and metastasis, has been validated using both in vitro and in vivo models of human OC omental metastasis [410]. In addition, ECM-mediated morphological changes in multicellular OC aggregates induce different properties that affect their ability to colonize secondary sites [440]. Recent research exhibits that HGSOC cells (OV90 and OVCAR3) often detach from tumor spheroids in clusters and are more resistant to anoikis. This implies that cell interactions may provide a survival advantage to these cells within clusters [441].

Integrin a2 facilitates OC cell adhesion to collagen, cell migration, unanchored cell growth, and mesothelial cell lining absence, causing peritoneal metastasis both in vitro and in vivo [439]. The initial steps of OC spherical structure formation may be affected by miRNAs, such as miR509-3p that acts through the Hippo pathway-yes1-associated transcriptional regulator (YAP)/ECM axis. For instance, miR-509-3p disrupts the migration and spherical structure of OVCAR8, a cell line with high YAP protein expression. Hence, the miR-509-3p/YAP1/ECM axis could be a potential treatment target for OCs with high YAP1 expression [442].

Ultimately, the complex procedure of progressive metastasis heavily relies on the critical interactions between OCs and stromal cells within the peritoneal microenvironment. OC cells adapt the metastatic site for their survival and spread by altering the ECM in the TME or inducing tumor-promoting changes in stromal cells. Simultaneously, stromal cells aid in the expansion and development of OC cells in the peritoneal cavity by encouraging new blood vessel formation, assisting tumor cell immune evasion and intrusion. Comprehensive understanding of the close interactions between cancer cells and the peritoneal microenvironment is essential for the formulation of effective therapeutic strategies.

## Current attempts to develop drugs targeting TME in OC

The TME has recently gained recognition as a key target for OC anti-tumor therapy. The TME is required for primary and metastatic growth and provides a target-rich niche for the development of promising anticancer drugs. Over the past decade, a variety of novel therapeutic strategies, including a range of targeted and immunological agents, have been introduced into routine clinical treatment plans, including poly (ADP-ribose) polymerase inhibitors (PARPi), immune checkpoint inhibitors (ICIs) and angiogenesis inhibitors [443, 444]. However, resistance to both chemotherapeutic agents and currently approved targeted therapies is common, while only a few OC patients respond to standalone ICIs immunotherapy, highlighting the difficulty in achieving complete remission of OC [445]. Therefore, given the aggressive nature of this tumor, it’s crucial to globally understand its biology to identify new clinical biomarkers and develop innovative treatments.

Extensive research on cancer immune interactions has led to improvements in the benefits of immunotherapy for cancer. Treatment with ICIs can counteract the immunosuppressive TME due to the high presence of immune checkpoint molecules on TILs and TALs [446]. Despite the clinical success of ICIs, such as Programmed Death Receptor-1(PD1)/ Programmed cell death ligand-1 (PD-L1) and cytotoxic T lymphocyte antigen-4 (CTLA-4), in treatment of some malignancies, only weak therapeutic responses have been observed in OC [447–449], which could be potentially attributed to the simultaneous presence of multiple immune checkpoint molecules. Recently, a study attempted to add a CTLA-4 blocking antibody during the initial TIL culture and found that CTLA-4 blockade favored the proliferation of CD8 + TIL in ovarian tumor fragments. Moreover, the addition of CTLA-4 blockade antibodies during the initial phase of TIL culture resulted in more effective anti-tumor TILs than standard TIL culture. This phenotype was maintained during the rapid expansion phase. These findings suggest that targeting CTLA-4 in the intact TME of tumor fragments can increase the number of TILs that respond to tumors, thereby improving clinical outcomes of TIL-based applied cell therapy (ACT) in OC [450]. Tumor immune combination therapies have achieved significant anti-tumor responses in patients compared to monotherapy. Novel combinations of PD-1/CTLA-4 with ICIs, such as Lymphocyte Activation Gene-3 (LAG-3) and mucin-domain-containing molecule-3 (TIM-3), have been shown to exert synergistic effects in preclinical OC models [451], thus providing a rationale for their therapeutic application. Another two related clinical trials have also achieved encouraging results (Table 1: NCT03365791 and NCT03099109).

**Table 1:** Selected examples of drug strategies in ongoing clinical trials that May target TME for the treatment of patients with ovarian cancer

NCT Number	Agents	Target	Mechanism of actions	Country and number of patients	Clinical trials	Recruiting Status	Brief summary and anticipated outcomes	
NCT04429542	BCA101 (+ Pembrolizumab)	CAFs	Tumor-Targeted Bifunctional Fusion Antibody for TGFB + EGFR (alone & combined with ICI)	United States; 292	Phase I	Recruiting	The study aims to evaluate the safety and tolerability of BCA101 monotherapy and in combination therapy in patients with EGFR-driven advanced solid tumors, including epithelial Ovarian Cancer. This bifunctional antibody may exert synergistic activity in patients with EGFR-driven tumors.	
NCT04969835	AVA6000	CAFs	FAP targeting for drug delivery	United States; 80	Phase I	Recruiting	This is a Phase 1, open label, dose-escalation and expansion study to evaluate the safety, pharmacokinetics and initial therapeutic activity of AVA6000, a Novel FAP-activated doxorubicin prodrug administered intravenously in patients with locally advanced or metastatic selected solid tumors.	
NCT04908787	BD0801 (+ Paclitaxel, Placebo, Topotecan, doxorubicin, liposome)	ECs	VEGF mAb (combined with chemotherapy)	China; 421	Phase III	Active, not recruiting	This is a randomized, double-blind, phase III study. Angiogenesis is critical for tumor growth and metastasis, and the VEGF/VEGF receptor signaling pathway is the most promising angiogenic target. The purpose of this study is to evaluate the efficacy and safety of BD0801 combination chemotherapy in patients with platinum-resistant recurrent OC.	
NCT02736305	Regorafenib	ECs	multi-TKI	Singapore; 21	Phase II	Completed	This is an open-label, single-arm phase 2 clinical trial. The objective of this study is to evaluate the efficacy and safety of regorafenib in Asian females with multiply recurrent OC.	
NCT04348032	PLD (+ Apatinib)	ECs	VEGFR inhibitor (combined with chemotherapy/ICI)	China; 152	Phase II	Active, not recruiting	This study is a randomized, parallel-controlled, multicenter clinical study. Angiogenesis is essential for advanced tumor growth and metastasis. And VEGF/VEGF receptor signaling pathway is the most promising angiogenic target due to its key roles in angiogenesis and tumor growth. This study sought to assess the efficacy and safety of the combination therapy of Apatinib and PLD, clarifying whether combination therapy could improve the outcomes of patients with platinum-resistant recurrent OC.	
NCT03797326	Pembrolizumab (+ Lenvatinib)	ECs	VEGFR inhibitor (combined with ICI)	United States; 590	Phase II	Ongoing	This is a multicenter, open-label phase 2 study. The purpose of this study is to determine the safety and efficacy of combination therapy with pembrolizumab and lenvatinib in in previously treated subjects with selected solid tumors.	
NCT04566952	Anlotinib (+ Olaparib)	ECs	multi-TKI (combined with PARPi)	China; 68	Phase II	Recruiting	This study is a single-arm, single-center, exploratory phase II study to investigate the efficacy and safety of anlotinib combined with dose-reduced olaparib as maintenance treatment in platinum-sensitive recurrent ovarian cancer patients.	
NCT02298959	Ziv-aflibercept (+ Pembrolizumab)	ECs	VEGF-trap (combined with ICI)	United States; 78	Phase I	Active, not recruiting	This phase I trial studies the side effects and best dose of ziv-aflibercept when given together with pembrolizumab in treating patients with advanced solid tumors. Ziv-afibercept works by decreasing blood and nutrient supply to the tumor, which may result in shrinking the tumor. Immunotherapy with monoclonal antibodies, such as pembrolizumab, may help the body’s immune system attack the cancer, and may interfere with the ability of tumor cells to grow and spread. Giving ziv-aflibercept together with pembrolizumab may be a better treatment for patients with advanced solid tumors.	
NCT00532194	Cediranib	ECs	Antiangiogenic VEGFR 1–3 inhibitor	United Kingdom; 486	Phase 3	Active, not recruiting	The purpose of this study is to assess the safety and efficacy of cediranib in combination with standard chemotherapy, in patients who have relapsed with ovarian, fallopian tube or epithelial cancer, after first line platinum-based treatment.	
NCT03170960	Cabozantinib (+ Atezolizumab)	ECs	multi-TKI (combined with ICI)	United States; 1732	Phase I	Recruiting	This is a multicenter Phase 1b, open-label study to assess safety, tolerability, preliminary efficacy, and pharmacokinetics of cabozantinib taken in combination with atezolizumab in subjects with multiple tumor types, including advanced OC.	
NCT01637532	Tocilizumab	Immune cells	An inhibitor of IL-6 receptor	Netherlands; 21	Phase I/II	Completed	The purpose of this interventional study is to determine the feasibility to combine standard chemotherapy (Carbo/Caelyx or doxorubicin) for recurrent ovarian cancer with immunotherapy (Tocilizumab and Peg-Intron).	
NCT02484404	Durvalumab & Olaparib & Cediranib	Immune cells	Anti-PD-L1, PARPi and VEGFR inhibitor	United States; 384	Phase I/II	Recruiting	This is a phase I/II study of the anti-programmed death ligand-1 antibody Durvalumab in Combination with Olaparib and/or Cediranib for advanced or recurrent solid tumors, including OC. This study has two components. In the phase 1 component of the study, researchers want to investigate how well participants tolerate the combination of these drugs in treating advanced solid tumors, and in the phase 2 part of this study, researchers want to study if the combination treatments are effective in OC.	
NCT03740165	Pembrolizumab & Olaparib	Immune cells	Anti-PD-1 and PARPi	United States; 1367	Phase III	Ongoing	This is a randomized phase 3, double-blind study. The purpose of this study is to assess the efficacy and safety of chemotherapy with or without Pembrolizumab followed by Maintenance with Olaparib or Placebo for the first-line treatment of BRCA Non-mutated advanced EOC.	
NCT03038100	Atezolizumab & Bevacizumab	Immune cells	Anti-PD-L1 and anti-angiogenic	United States; 1301	Phase III	Completed	This is a Phase III, global, double-blind, 2-arm randomized study designed to compare the efficacy and safety of atezolizumab + paclitaxel + carboplatin + bevacizumab versus placebo + paclitaxel + carboplatin + bevacizumab in newly-diagnosed Stage III or Stage IV OC.	
NCT05231122	CDX-1140 & Bevacizumab & Pembrolizumab	Immune cells	Agonistic anti-CD40 mAb potentiating APC functions	United States; 80	Phase II	Not yet recruiting	This phase II trial tests whether pembrolizumab combined with bevacizumab with or without agonist anti-CD40 CDX-1140 works to shrink tumors in patients with ovarian cancer that has come back (recurrent).	
NCT04503980		MSLN-CAR T cells secreting PD-1 nanobodies	Immune cells	Anti-PD-1 MSLN-directed CAR T cells	United States; 10	Phase I	Recruiting	This is a single arm, open-label, dose escalation clinical study to evaluate the safety and tolerability of autologous mesothelin (MSLN)-targeted chimeric antigen receptor (MSLN-CAR) T cells secreting PD-1 nanobodies (αPD1-MSLN-CAR T cells) in patients with solid tumors, including OC.
NCT02042430	Epacadostat	Immune cells	IDO inhibitor restoring the activation of immune cell	United States; 17	Phase I	Active, not recruiting	This pilot Early Phase I clinical trial studies epacadostat before surgery in treating patients with newly diagnosed stage III-IV epithelial ovarian, fallopian tube, or primary peritoneal cancer.	
NCT04611126	Ipilimumab & Relatlimab& Nivolumab	Immune cells	Anti-CTLA-4, anti-LAG-3 and anti-PD-1	Denmark;18	Phase I/II	Recruiting	The study aims to demonstrate that ACT and a combination of Relatlimab-Nivolumab does not increase the toxicity compared to the same treatment regimen including Nivolumab monotherapy. The study elucidates whether the combination Relatlimab-Nivolumab lead to objective responses and improves PFS. It is anticipated that combining Relatlimab and Nivolumab with ACT for advanced OC is safe and feasible.	
NCT02159716	CART-meso	Immune cells	MSLN-directed CAR T cells	United States; 19	Phase I	Completed	This is a phase I study to establish safety and feasibility of intravenously administered lentiviral transduced CART-MESO cells administered with and without cyclophosphamide in a 3 + 3 dose escalation design in patients with metastatic pancreatic cancer, serous epithelial ovarian cancer, or pleural mesothelioma.	
NCT02764333	Durvalumab & TPIV200/ huFR1	Immune cells	Anti-PD-L1 combined with multi-epitope anti-folate receptor vaccine	United States; 29	Phase II	Completed	This is a Phase 2 clinical trial, which tests two investigational drugs: TPIV200/huFR-1, which is a vaccine consisting of proteins from the folate receptor alpha mixed with GM-CSF, and durvalumab, which is an antibody drug that help un-block parts of the immune system. The aim of this study is to find out the safety and efficacy of combination drug therapy in patients with platinum resistant OC.	
NCT02650986	TGFbDNRII-transduced autologous TILs & Decitabine	Immune cells	TCR therapy targeting NY- ESO-1 & hypomethylation agent	United States; 15	Phase I/II	Ongoing	This is a phase I/IIa, dose-escalation study of NY-ESO-1 TCR/TGFbDNRII-transduced TILs. This study evaluates the side effects and best dose of gene-modified T cells when given with or without decitabine, and to see how well they work in treating patients with advanced malignancies expressing NY-ESO-1.	
NCT01772004	Avelumab	Immune cells	Anti-PD-L1	United States; 1756	Phase I	Completed	This is a Phase 1, open-label, dose-escalation trial of avelumab [antibody targeting programmed death ligand 1 (anti PD-L1) in participants with selected tumors, including OC.	
NCT03099109	LY3321367(+ LY3300054)	Immune cells	Anti-TIM-3 and Anti-PD-L1	United States; 209	Phase Ia/Ib	Completed	This clinical study evaluated a novel, first-in-class TIM-3 monoclonal antibody, LY3321367, alone or in combination with the anti-PD-L1 antibody, LY300054, in patients with advanced solid tumors, including OC. This open-label, multicenter, phase Ia/b study was designed to determine the safety/tolerability and recommended phase II dose of LY3321367 with or without LY300054.	
NCT03365791	Spartalizumab and ieramilimab	Immune cells	Anti-PD -1 and Anti-LAG-3	United States; 76	Phase II	Completed	This is a phase II, open-label study to determine the efficacy and safety of the combination of spartalizumab and ieramilimab in multiple tumor types (including OC) that have relapsed and/or are refractory to available standard of care therapies.	
NCT05225363	TAG72-CAR-T cells, Cyclophosphamide and Fludarabine	Immune cells	anti-TAG72 CAR antibodies	United States; 33	Phase I	Recruiting	This phase I trial tests the safety, side effects, and best dose of TAG72-CAR T cells in treating patients with epithelial OC that remains despite treatment with platinum therapy (platinum resistant).	
NCT02498912	Cyclophosphamide and Genetically-modified T cells	Immune cells	Anti-MUC16ecto tumor antigen	United States; 18	Phase I	Active, not recruiting	The purpose of this phase I study is to test the safety of different dose levels of modified T cells, in which the patients’ own T cells are genetically modified to target the MUC16ecto tumor antigen and secrete IL-12, overcome the inhibitory effects of the solid TME, promote the proliferation of infused CAR-T cells, and enhance the immune response at the tumor site.	
NCT05672459	IVS-3001	Immune cells	Anti-HLA-G CAR-T cells	United States; 117	Phase I/ IIa	Recruiting	The proposed clinical study is a Phase 1/2a trial to investigate the safety, tolerability, pharmacokinetics and clinical activity of anti-HLA-G CAR-T cells IVS-3001 administered to subjects with previously treated, locally advanced, or metastatic solid tumors.	
NCT05518253	CD70 CAR-T cells	Immune cells	CD70 + CAR-T cells	China; 18	Phase I	Recruiting	This is a phase I, single-center, two-arm, open-label study to evaluate the safety and tolerability of CAR-T in patients with advanced/metastatic solid tumors that are CD70 positive, and to obtain the maximum tolerated dose and phase II recommended dose of CAR-T.	
NCT03638206	CAR-T/TCR-T cells	Immune cells	anti-C-MET antibody	China; 73	Phase I-II	Recruiting	This is a single-arm, open-label, single-center, phase I-II study of a multi-targeted genetically modified immunotherapy. The aim is to evaluate the safety and efficacy of CAR-T/TCR-T cell immunotherapy in patients with different malignancies.	
NCT04627740	Retroviral vector-transduced autologous T cells	Immune cells	Anti-ALPP CART-cells	China; 20	Phase I/II	Recruiting	This is a single-arm, single-center, open-label pilot study of anti-ALPP CAR-T cells in patient with ALPP-positive advanced solid tumor. The goal is to evaluate the safety and efficacy of anti-ALPP CAR-T cells in treating these patients.	
NCT03585764	MOv19-BBz CAR-T cells	Immune cells	Folate receptor-α-directed CAR T cells	United States; 18	Phase I	Recruiting	Phase I study to establish safety and feasibility of intraperitoneally administered lentiviral transduced MOv19-BBz CAR T cells with or without cyclophosphamide + fludarabine as lymphodepleting chemotherapy	
NCT04660929	CT-0508	Immune cells	CAR macrophages targeting HER2 + tumor cells	United States; 48	Phase I	Recruiting	This is a phase 1, first-in-human, open label study of CAR macrophages in HER2 overexpressing solid tumors.	
NCT03916679	Anti-MESO CAR-T cells	Immune cells	MSLN-directed CAR T cells	China; 20	Phase I/II	Recruiting	Phase I/II clinical trials aim to study the feasibility and efficacy of anti-MESO antigen receptors T cell therapy for relapsed and refractory EOC.	
NCT05239143	PD1-MUC16- CAR-T cells	Immune cells	CAR-T cell therapy	United States; 180	Phase I	Recruiting	This is a Phase 1, open label, dose escalation and expanded cohort study of P-MUC1C-ALLO1 in adult subjects with advanced or metastatic epithelial derived solid tumors, including OC. The goal is to discuss the safe dose, safety and tolerability of this CAR-T cell therapy.	
NCT04691375	PY314 (+ Pembrolizumab)	Immune cells	Anti-TREM2 mAb depleting TREM2 + TAMs	United States; 288	Phase I	Active, not recruiting	This is a multicenter, a Phase 1a/1b open-label study to evaluate the safety, tolerability, pharmacokinetics and pharmacodynamics of PY314 as a single agent and in combination with pembrolizumab in subjects with advanced solid tumors.	
NCT04881045	PF-07257876	Immune cells	Anti-CD47/PD-L1 bispecific mAb	United States; 28	Phase I	Active, not recruiting	This is a first-in-human, Phase 1, open label, multicenter, multiple dose, dose escalation and dose expansion study intended to evaluate the safety, pharmacokinetic, pharmacodynamic and potential clinical benefit of PF-07257876, a CD47-PD-L1 bispecific antibody, in participants with selected advanced or metastatic tumors for whom no standard therapy is available.	
NCT04670068	CAR.B7-H3	Immune cells	B7-H3-directed CAR T cells	United States; 21	Phase I	Recruiting	This is single center, open-label phase 1 dose escalation trial. The purpose is to test the safety of using a new treatment called autologous T lymphocyte chimeric antigen receptor cells against the B7-H3 antigen (CAR.B7-H3 T cells) in patients with recurrent EOC.	
NCT05403554	NI-1801	Immune cells	Anti-CD47/mesothelin bispecific mAb	France; 40	Phase I	Recruiting	This is an open-label, Phase 1, dose escalation and expansion, first-in-human clinical study of NI-1801 in subjects with advanced, metastatic, or recurrent solid malignancies expressing mesothelin.	
NCT05261490	Maplirpacept + PLD	Immune cells	Fusion protein blocking CD47	United States; 11	Phase I/II	Active, not recruiting	The purpose of this study is to assess maplirpacept (PF-07901801) administered in combination with PLD in patients with platinum-resistant ovarian cancer and for whom PLD is a reasonable treatment option.	
NCT03692637	Mesothelin Car NK-HNRM- 01	Immune cells	Anti-MSLN CAR NK cells	United States; 30	Phase I	Not yet recruiting	This is a single centre、single arm、open-label, to investigate the safety and efficacy of anti-Mesothelin Car NK Cells With OC.	
NCT03602859	Niraparib + Pembrolizumab	Immune cells	Anti-PD-1 and PARPi	United States; 1402	Phase III	Active, not recruiting	This is a global, multicenter, randomized, double-blind, controlled Phase 3 study that will primarily compare the PFS for participants receiving dostarlimab with standard of care chemotherapy +/- bevacizumab followed by niraparib and dostarlimab maintenance +/- bevacizumab versus participants receiving standard of care with chemotherapy followed by niraparib maintenance.	
NCT03522246	Olaparib + Bevacizumab	Immune cells	PARPi and VEGFR inhibitor	United States; 1097	Phase III	Active, not recruiting	This is a Phase 3, randomized, multinational, double-blind, dual placebo-controlled, 4-arm study evaluating rucaparib and nivolumab as maintenance treatment following response to front-line treatment in newly diagnosed OC patients. Response to treatment will be analyzed based on homologous recombination status of tumor samples.	
NCT03740165	Pembrolizumab + Olaparib	Immune cells	Anti-PD-1 and PARPi	United States; 1367	Phase III	Active, not recruiting	This is a Randomized Phase 3, Double-Blind Study of Chemotherapy With or Without Pembrolizumab Followed by Maintenance With Olaparib or Placebo for the First-Line Treatment of BRCA Non-mutated Advanced OC. Primary endpoints are investigator-assessed PFS and OS.	
NCT03737643	Rucaparib + Atezolizumab	Immune cells	Anti-PD-L1 and PARPi	United States; 1407	Phase III	Active, not recruiting	This is a Phase III randomized, double-blind, multi-centre study to evaluate the efficacy and safety of durvalumab in combination with standard of care platinum-based chemotherapy and bevacizumab followed by maintenance durvalumab and bevacizumab or durvalumab, bevacizumab and olaparib in patients with newly diagnosed advanced OC.	
NCT03287271	VS-6063 (+ Carboplatin, Paclitaxel)	ECM (-cell interactions)	FAK inhibitor (+ chemotherapy)	United States; 90	Phase I/II	Recruiting	The purpose of the study is to investigate the efficacy and safety of combination VS-6063, carboplatin, and paclitaxel. in the treatment of patients with OC.	
NCT03078400	SPL-108 (+ Paclitaxel)	ECM (-cell interactions)	CD44 antibody blocking peptide (+ chemotherapy)	United States; 14	Phase I	Ongoing	This is an open-label phase 1 trial that aims to evaluate the safety and efficacy of daily subcutaneous SPL-108 injections when used in combination with paclitaxel in patients with platinum-resistant, CD44+, advanced EOC.	
NCT03917043	APG-2449	ECM (-cell interactions)	ALK/ROS1/FAK inhibitor	China; 150	Phase I	Recruiting	APG-2449 is a novel, orally active, multi-targeted tyrosine kinase inhibitor, which inhibits FAK, ALK, and ROS1 with nanomolar potencies. This phase I study aims to evaluate the safety, pharmacokinetic and pharmacodynamic properties of orally administered APG-2449 in patients with advanced solid tumors, including OC.	
NCT03875820	VS-6766 & VS-6766	ECM (-cell interactions)	RAS/MEK inhibitor and FAK inhibitor	United Kingdom;87	Phase I	Active, not recruiting	This is a multi-center, investigator-initiated, dose escalation, Phase I trial of the safety, tolerability and the pharmacodynamic activity of combination of the FAK inhibitor, VS-6063, and the dual RAF/MEK inhibitor, VS-6766 in patients with advanced solid tumors, including OC.	
NCT03564340	Ubamatamab (REGN4018)	CAMs	Human bispecific antibody targeting MUC16 and CD3	United States; 690	Phase I/II	Recruiting	This is a phase 1/2 study of REGN4018, a MUC16xCD3 bispecific antibody, alone or in combination with cemiplimab in patients with recurrent OC. The objective of this study is to investigate the safety and pharmacokinetics of REGN4018 in the treatment of recurrent advanced OC, as well as the safety and tolerability of pretreatment in combination with cemiplimab.	
NCT04938583	Oregovomab + Bevacizumab + Paclitaxel + Carboplatin	CAMs	Anti-MUC16, VEGFR inhibitor, (+ chemotherapy)	Korea; 54	Phase I/II	Recruiting	This study is an open-label, single arm, phase 1b/II, multicenter study. The study aims to evaluate the safety and activity of Oregovomab and Bevacizumab, Paclitaxel Carboplatin as a combinatorial strategy in subjects with BRCA-wild type platinum sensitive recurrent OC.	
NCT01039207	Rilotumumab	CAMs	A inhibitor of HGF: HGF/MET pathway	United States; 31	Phase II	Completed	This phase II trial studies how well Rilotumumab works in treating patients with recurrent or persistent OC.	
NCT00635193	Volociximab + Liposomal	CAMs	Anti-angiogenic integrin inhibitor (+ chemotherapy)	United States; 138	Phase I/II	Completed	This is an open-label study of liposomal doxorubicin with or without volociximab for the treatment of subjects with advanced epithelial ovarian cancer or primary peritoneal cancer relapsed after prior therapy with Plat/Taxane-based chemo.	
NCT03587311	Anetumab Ravtansine + Bevacizumab + Paclitaxe	CAMs	Anti-MSLN, VEGFR inhibitor, (+ chemotherapy)	United States; 96	Phase I	Active, not recruiting	This phase II trial studies the side effects of bevacizumab and anetumab ravtansine or paclitaxel in treating patients with ovarian, fallopian tube, or primary peritoneal cancer that does not respond to treatment (refractory).	
NCT00325494	MORAb-009	CAMs	Anti-MSLN	United States; 24	Phase I	Completed	This clinical trial is being performed to determine the safety of MORAb-009 in subjects with mesothelin-expressing tumors, as well as to establish serum pharmacokinetics of the antibody, and to assess tumor antigens that may serve as predictors of a response to MORAb-009.	

**Abbreviation**: ACT, adoptive cell therapy; CAR, chimeric antigen receptor; ECs, endothelial cells; CAFs, cancer associated fibroblasts; ECM, extracellular matrix; EGFR: epidermal growth factor receptor; FAP: fibroblast activation protein; FAK: focal adhesion kinase; ICI: immune checkpoint inhibitor; mAb: monoclonal antibody; MSLN: mesothelin; MUC: mucin; PFS: progression free survival; PLD, Pegylated liposomal doxorubicin; RAS: rat sarcoma virus; TCR: T cell receptor; TILs: tumor infiltrating lymphocytes; TKI: tyrosine kinase inhibitors; TAM, tumor-associated macrophage; TREM2, triggering receptor expressed on myeloid cells 2; IDO, indoleamine 2,3-dioxygenase; ALPP, Alkaline Phosphatase, Placental; CD47, Cluster of Differentiation 47; CD70, Cluster of Differentiation 70; CTLA-4, Cytotoxic T-Lymphocyte-Associated Protein 4; GM-CSF, Granulocyte-Macrophage Colony-Stimulating Factor; HGF, Hepatocyte Growth Factor; HLA-G, Human Leukocyte Antigen-G; IL-6, Interleukin-6; LAG-3, Lymphocyte-Activation Gene 3; MET, Mesenchymal-Epithelial Transition factor; OS, overall survival; PD-1, Programmed Cell Death Protein 1; PD-L1, Programmed Death-Ligand 1; TIM-3, T-cell Immunoglobulin and Mucin domain-containing protein 3; VEGF, Vascular Endothelial Growth Factor; VEGFR, Vascular Endothelial Growth Factor Receptor

The effectiveness of ICIs in OC is hindered by the absence of tumor-reactive TILs and the loss of HLA-mediated antigen presentation by tumor cells, preventing the initiation of a targeted immune response even when TILs and TALs are activated [147]. Recent research shows that neoadjuvant chemotherapy significantly changes the expression of immunosuppressive molecules. This implies that ICI combinations should be customized based on the immunological TME composition post-neoadjuvant chemotherapy [452]. In addition, TILs and TALs used in ACT have demonstrated success in some cancer types. ACT with chimeric antigen receptor (CAR) T cells is a promising therapeutic approach for advanced OC. CAR redirects T cell specificity and function to recognize tumor antigens independently of HLA and fully activates the effector function of T cells. The CAR targets currently used for OC therapy include MSLN, MUC-1 and B7-H3 [453]. CAR-T therapy has not yet been extensively studied in clinical trials for OC patients, but studies have yielded positive results in their treatment. One trial is currently underway for patients with recurrent/resistant OC who have progressed on two prior therapies using follicle-stimulating hormone receptor (FSHR T)-mediated T cells (NCT05316129). Another ongoing trial (NCT04670068) aims to evaluate the efficacy of CAR-T cells with the B7-H3 antigen in recurrent OC, bringing new treatment hopes to refractory OC patients. Furthermore, Researchers at City of Hope Medical Center are currently conducting a first-in-human phase 1 trial (NCT05225363) to verify the safety and efficacy of a CAR-T cell therapy targeting tumor-associated glycoprotein-72 (TAG-72, a protein found on the surface of OC cells). This therapy can produce significant anti-tumor efficacy in mouse models, with a complete response rate of 40%. The trial included patients with advanced OC who had previously received platinum-based chemotherapy, and it was confirmed in the laboratory and preclinical models that TAG72-CAR T Cells therapy may be able to eradicate OC cells. Other clinical trials currently underway using CAR-T cell therapy for OC are summarized in Table 1 (NCT04627740, NCT03638206, NCT05518253, NCT05672459, NCT02498912). However, the application of CAR-T cell therapy in OC presents several challenges. The substantial intratumoral and intertumoral heterogeneity characteristic of OC complicates the ability of a single CAR-T cell to uniformly target al.l tumor cells. Tumor cells may evade immune detection through mechanisms such as antigen loss or downregulation, contributing to immune escape. Additionally, meticulous attention must be directed towards minimizing both on-target and off-tumor CAR-T cell-mediated toxicity, given the potential expression of target antigens on non-cancerous cells.

Three PARP inhibitors—olaparib, niraparib, and rucaparib—have been approved for use as maintenance therapies under various clinical conditions [454]. Following the identification of PARP inhibitors as targeted treatments for OC, the principle of synthetic lethality has been employed. This approach involves inducing cancer cell death by exploiting defects in homologous recombination repair, such as BRCA1/2 mutations, and concurrently inhibiting the DNA damage response pathway with PARP inhibitors. The utilization of PARP inhibitors in conjunction with chemotherapy presents challenges due to their overlapping toxicity profiles. Consequently, a strategy has emerged that focuses on delaying or preventing disease progression through sequential use and long-term maintenance. Specifically, the application of PARP inhibitors following first- and second-line platinum-based chemotherapy responses has been shown to extend the interval between therapeutic response and disease recurrence. The most robust evidence indicates that the early incorporation of PARP inhibitors in first-line therapy may facilitate time-limited maintenance treatment and potentially achieve a cure in certain patients, as demonstrated by the lack of relapse following drug discontinuation [455].

Immunotherapy can only succeed in treating HGSOC if it targets multiple aspects of the TIME and ECM of OC, given their complexity. However, multiple combination therapies have had limited success to date, including ICI with chemotherapy [456–458] and ICI with anti-angiogenic therapy [444]. Further exploration of combination therapy is therefore necessary for rational, multifactorial targeting of the TME in OC. An ongoing phase III clinical trial for OC (NCT03740165) is a randomized, double-blind phase III clinical study that included a total of 1,367 patients. The aim of this study is to evaluate the efficacy and safety of pembrolizumab in combination with chemotherapy (paclitaxel and carboplatin), followed by maintenance therapy with pembrolizumab and olaparib (with or without bevacizumab) as a first-line treatment option for patients with advanced OC with a non-mutated BRCA status. The primary endpoints were PFS in patients with combined positive score ≥ 10 of PD-L1 expression and PFS in the intent-to-treat population. Some of the results showed that compared with chemotherapy alone, the PFS of patients in the pembrolizumab plus olaparib group was significantly improved, which was of statistical and clinical significance. In addition, several other phase III clinical trials are still ongoing to explore the effectiveness and rationality of the combination of ICI and PARPi (Table 1: NCT03602859; NCT03522246; NCT03737643).

Advances in nanoscience have brought new opportunities for the diagnosis and treatment of OC [459]. Nanoparticles (NPs) can modulate the OC immune TME by stimulating the immune response of M1-TAMs, DCs, and T cells, while reducing the infiltration of immunosuppressive cells such as M2-TAMs and Tregs. To date, a variety of nanomedicines have been approved for clinical treatment of OC, including doxorubicin hydrochloride liposomes [460, 461] and albumin paclitaxel [462]. The inherent properties of NPs that preferentially localize to tumor tissue and cells in the TME not only help to reduce systemic toxicity [460], but also enhance the anti-tumor effect by increasing the permeability and retention of tumor tissue [463]. It is gratifying to note that nanotechnology combined with intraperitoneal administration techniques has been shown to have a strong inhibitory effect on OC metastasis, given the characteristics of OC, which has extensive metastasis in the pelvic and abdominal cavities [464]. In addition, some studies have found that epigenetic changes, including DNA methylation and histone modifications, are being characterized in OC and functionally linked to processes related to OC occurrence, chemoresistance, cancer stem cell survival and metastasis have been functionally linked [465–467]. DNA methylation and histone modifications are reversible, and epigenome-targeted therapies may help to improve the immunosuppressive state of the TME. Excitingly, various epigenetics-based combination therapies have been shown to have significant antitumor effects, and these combinations may be potential therapeutic strategies for OC [468, 469].

Other strategies are being developed to target non-immune cells involved in fibrotic response, immunosuppressive microenvironment formation, and ECM-cancer cell communication in OC. The ongoing clinical trials targeting the TME in OC are summarized in Table 1. The roles and interactions of the TME, stroma, ECM, and related receptors in various disease stages need more detailed explanation. Although novel therapeutic approaches targeting the TME do not cure OC, these strategies have the potential ability to limit its progression and are expected to eventually lead to groundbreaking insights and lower patient mortality. We believe that TME-targeting strategies should be employed as a valuable adjuvant therapy for OC.

## Conclusions and future perspectives

There is growing evidence that the TME is closely associated with the development, progression, and metastasis of OC. Extensive intercellular communication and signaling exists between OC cells and surrounding stromal cells. Therefore, exploring OC from the perspective of the TME may provide new insights and potential therapeutic targets. This review comprehensively discusses the mechanisms by which key components of the TME contribute to the development, drug resistance, and metastasis of OC. It also summarizes current attempts to develop therapies targeting the TME in OC, including CAF-targeted therapies, anti-angiogenic agents, TAM-targeted treatments, ICIs, and chemokine inhibitors. These therapies have either received clinical approval or are currently under investigation.

However, although this review systematically summarizes the role of TME in OC generation and metastasis, the following key areas still require in-depth exploration: First, the association between TME molecular heterogeneity and treatment response is insufficient. Current functional analyses of TME components are mostly based on the whole population, but the molecular characteristics of TME in different OC subtypes and their impact on platinum resistance remain unclear. In addition, most studies rely on traditional 2D cell lines or Patient-Derived tumor Xenograft models, which fail to simulate the spatiotemporal dynamics of the TME in peritoneal metastasis (e.g., metabolic interactions between adipocytes and tumor cells). Third, the response mechanism of immunotherapy is unknown. Although ICIs (e.g., PD-1 inhibitors) are effective in some patients, the overall response rate is low. The synergistic effects of multiple immunosuppressive signals in the TME (e.g., IL-10, CCL22) and the mechanism of antigen presentation defects need to be further elucidated.

Therefore, future research should focus on the following aspects. First, combining single-cell transcriptomics, spatial metabolomics, and proteomics technologies to elucidate the spatial and temporal heterogeneity of TME components (e.g., the polarization status of TAMs and CAF subsets) and establish a molecular typing framework to guide individualized targeted therapy (e.g., CSF-1R inhibitors targeting M2-TAMs). Second, a 3D organoid co-culture system or microfluidic chip will be developed to integrate peritoneal mesothelial cells, adipocytes and immune cells to simulate the dynamic interactions in the pre-metastatic niche and screen for combination therapies targeting the TME-tumor interaction (e.g., anti-VEGF + anti-IL-6). Third, the remodeling of the immune microenvironment will be explored in depth. Investigate the co-expression patterns of multiple checkpoint molecules (e.g., LAG-3, TIM3) in the TME, and design bispecific antibodies or epigenetic regulators to reverse T cell exhaustion and enhance DC antigen presentation. In addition, to optimize clinical effectiveness, combination therapies should be strategically developed using patient-specific tumor data, genomic analyses, molecular assays, and new predictive and prognostic biomarkers. This will aid in selecting suitable drug candidates for personalized cancer treatment.

In conclusion, OC remains a lethal malignancy characterized by insidious onset, early metastasis, and high recurrence rates post-treatment. The mechanisms underlying the biology and aggressiveness of OC largely remain to be elucidated. A deeper understanding of the role of the TME in supporting the growth, progression and metastatic spread of OC cells, supported by technological advances, will provide an untapped resource of anti-tumor targets, ushering in a new era of precision medicine.

## Data Availability

No datasets were generated or analysed during the current study.

## Abbreviations

OC: Ovarian cancer
TME: Tumor microenvironment
HGSOC: High grade serous OC
ECM: Extracellular matrix
TIME: Tumor Immune Microenvironment
TAMs: Tumor-associated macrophages
CSF-1: Colony-stimulating factor-1
IL: Interleukin
TGF-β: Transforming growth factorβ
TNF-α: Tumor necrosis factorα
CCL18: C-C chemokine motif ligand 18
OS: Overall survival
PFS: Progression Free Survival
Tregs: Regulatory T cells
MMPs: Matrix metalloproteinases
VEGF: Vascular endothelial growth factor
POSTN: Periostin
IGF-1: Insulin-like growth factor-1
MUC2: Mucin 2
TANs: Tumor-associated neutrophils
JAG2: Jagged2
NLR: Neutrophil-to-lymphocyte ratio
MDSCs: Myeloid-derived suppressor cells
PGE2: Prostaglandin E2
TILs: Tumor-infiltrating lymphocytes
TALs: Tumor-associated lymphocytes
CXCR2: C-X-C Motif Chemokine Receptor 2
DCs: Dendritic cells
APCs: Antigen-presenting cells
cDCs: Conventional DCs
pDCs: Plasmacytoid DCs
IFNγ: Interferon-gamma
PD-L1: Programmed death-ligand 1
COX2: Cyclooxygenase 2
NK cells: Natural killer cells
CAAs: Cancer-associated adipocytes
LEP: Leptin
FABP4: Fatty acid-binding protein 4
CAFs: Cancer-associated fibroblasts
CTHRC1: Collagen triple helix repeat-containing-1
COL11A1: Collagen type XI alpha 1
EGF: Epidermal growth factor
VCAN: Versican
Hh: Hedgehog
FGF-1: Fibroblast growth factor-1
HGF: Hepatocyte growth factor
DKK3: Dickkopf-3
FMO2: Flavin-containing monooxygenase 2
TAECs: Tumor-associated endothelial cells
EZH2: Enhancer of Zeste Homolog 2
MIF: Migration inhibitory factor
HIF-1: Hypoxia-inducible factor-1
CAMs: Cancer-associated mesothelial cells
LPA: Lysophosphatidic acid
MMT: Mesothelial-mesenchymal transition
HA: Hyaluronic acid
MDR1: Multidrug resistance-1
ABC: ATP binding cassette
FN: Fibronectin
CA-MSCs: Cancer-associated mesenchymal stem cells
ADSCs: Adipose-derived mesenchymal stem cells
SMAD: Small mother against decapentaplegic
EpCAM: Epithelial cell adhesion molecule
pGSN: Plasma gelsolin
pGSN: Exosomes with plasma gelsolin
eNK-EXO: Exosomes derived from expanded natural killer cells
PDCD4: Programmed cell death gene 4
uPA: Urokinase-type plasminogen activator
KLK: Kallikrein-related peptidase
PDK1: Pyruvate dehydrogenase kinase isoform 1
ATCs: Ascites tumor cells
DDR2: Discoidin domain receptor 2
ASK1: Apoptosis signal-regulated kinase 1
N1ICD: Notch1 receptors
ITLN1: Intelectin-1
LTF: Lactotransferrin
LRP1: Lipoprotein receptor-related protein 1
iPLA2: Calcium-independent phospholipase A2
cPLA2: Cell membrane phospholipase A2
YAP: Yes1-associated transcriptional regulator
PARPi: Poly (ADP-ribose) polymerase inhibitors
ICIs: Immune checkpoint inhibitors
ACT: Applied cell therapy
CAR: Chimeric antigen receptor
PD1: Programmed Death Receptor-1
PD-L1: Programmed cell death ligand-1
CTLA-4: cytotoxic T lymphocyte antigen-4
LAG-3: Lymphocyte Activation Gene-3
TIM-3: mucin-domain-containing molecule-3
FOXP3: Forkhead box P3
